# Beyond the surface: exploring the mycobiome of Norway spruce under drought stress and with *Heterobasidion parviporum*

**DOI:** 10.1186/s12866-023-03099-y

**Published:** 2023-11-17

**Authors:** Blessing Durodola, Kathrin Blumenstein, Adedolapo Akinbobola, Anna Kolehmainen, Victor Chano, Oliver Gailing, Eeva Terhonen

**Affiliations:** 1 Forest Pathology Research Group, Büsgen-Institute, Department of Forest Botany and Tree Physiology, Faculty of Forest Sciences and Forest Ecology, University of Göttingen, Büsgenweg 2, 37077 Göttingen, Germany; 2 Department of Forest Genetics and Forest Tree Breeding, Büsgen-Institute, Faculty of Forest Sciences and Forest Ecology, University of Göttingen, Büsgenweg 2, 37077 Göttingen, Germany; 3 Chair of Pathology of Trees, Institute of Forestry, Faculty of Environment and Natural Resources, University of Freiburg, Bertoldstr. 17, 79098 Freiburg, Germany; 4 Department of Cell Biology, Centre for Organismal Studies, University of Heidelberg, Im Neuenheimer Feld 230, 69120 Heidelberg, Germany; 5 Natural Resources Institute Finland (Luke), Forest Health and Biodiversity, Latokartanonkaari 9, 00790 Helsinki, Finland

**Keywords:** *Picea abies*, Plant-host relationship, Drought stress, Genotypic variation, Microbes, Fungal community, Environmental conditions

## Abstract

The mycobiome, comprising fungi inhabiting plants, potentially plays a crucial role in tree health and survival amidst environmental stressors like climate change and pathogenic fungi. Understanding the intricate relationships between trees and their microbial communities is essential for developing effective strategies to bolster the resilience and well-being of forest ecosystems as we adopt more sustainable forest management practices. The mycobiome can be considered an integral aspect of a tree’s biology, closely linked to its genotype. To explore the influence of host genetics and environmental factors on fungal composition, we examined the mycobiome associated with phloem and roots of Norway spruce (*Picea abies* (L.) Karst.) cuttings under varying watering conditions. To test the “mycobiome-associated-fitness” hypothesis, we compared seedlings artificially inoculated with *Heterobasidion parviporum* and control plants to evaluate mycobiome interaction on necrosis development. We aimed to 1) identify specific mycobiome species for the Norway spruce genotypes/families within the phloem and root tissues and their interactions with *H. parviporum* and 2) assess stability in the mycobiome species composition under abiotic disturbances (reduced water availability). The mycobiome was analyzed by sequencing the ribosomal ITS2 region. Our results revealed significant variations in the diversity and prevalence of the phloem mycobiome among different Norway spruce genotypes, highlighting the considerable impact of genetic variation on the composition and diversity of the phloem mycobiome. Additionally, specific mycobiome genera in the phloem showed variations in response to water availability, indicating the influence of environmental conditions on the relative proportion of certain fungal genera in Norway spruce trees. In the root mycobiome, key fungi such as *Phialocephala fortinii* and *Paraphaeosphaeria neglecta* were identified as conferring inhibitory effects against *H. parviporum* growth in Norway spruce genotypes. Furthermore, certain endophytes demonstrated greater stability in root ecosystems under low water conditions than ectomycorrhizal fungi. This knowledge can contribute to developing sustainable forest management practices that enhance the well-being of trees and their ecosystems, ultimately bolstering forest resilience.

**Supplementary Information:**

The online version contains supplementary material available at 10.1186/s12866-023-03099-y.

## Introduction

The hidden mycobiome of ecosystems may represent one of the key solutions for increasing resilience in forest trees during climate change. In the field of evolutionary ecology, the “insurance hypothesis” proposes that a wide variety of species maintains the cohesion of an ecosystem while there are alterations in both biotic and abiotic environmental conditions [1–5]. Similarly, competitive exclusion ensures that beneficial fungi have the potential to overcome pathogens in the same habitat [6, 7]. In the plant roots, dark septate endophytes (DSE) can improve water balance and increase resistance to drought [8], and foliar endophytes have been shown to protect their host against fungal pathogens [9] and pests [10–12]. In that sense, diverse mycobiomes can act as part of tree resistance, enabling trees to respond to new stress [13]. We hypothesize that the hidden mycobiome biodiversity provides a link between more diverse mycobiomes and certain biological processes (function) needed to increase tree resilience (fewer disease symptoms/more adapted to environmental stress). We term this the “mycobiome-associated-fitness” hypothesis. This kind of extended tree resistance can be crucial in the near future as latent pathogens/saprotrophs can switch from symptomatic to a pathogenic lifestyle under abiotic disturbance, for instance, drought [14, 15].

One of the important pathogens of Norway spruce (*Picea abies* (L.) Karst.) is the species complex *H. annosum* sensu lato, a group of fungi that cause root rot and stem decay [16, 17]. *Heterobasidion parviporum* Niemelä & Korhonen belongs to the *H. annosum* species complex recognized in Europe and is associated with Norway spruce. However, it can also be associated with some other conifers, such as *Abies* and *Pinus* species [16]. The primary infection by *Heterobasidion* species takes place when the windborne basidiospores are deposited onto fresh stump surfaces or wounds on the tree stem and roots. *H*. *parviporum* can continue spreading through root networks and infect healthy neighbouring trees [16]. The continuous threat of root and stem rot to Norway spruce leads to severe economic losses to the forest industry. There is currently no treatment available for infected trees, and the control strategies for this fungus can only be implemented after the harvest, and there are no measures in place for dealing with it in living trees.. Further, selecting more tolerant trees through breeding could improve resistance as Norway spruce genotypes vary in their susceptibility to *H*. *parviporum* [18]. The host plant genotype has been noted to be also one factor defining the fungal endophytic composition [19–22]. In that sense, the mycobiome is an integral part of a tree’s overall biology and can be seen as an extension of its genotype. Similarly, a few Norway spruce root fungal endophytes, such as *Phialocephala sphaeroides,* have been noted to suppress *H*. *parviporum* growth [23, 24] and *Phytophthora citricola* s.l. [25] under in vitro conditions. It can be hypothesized that choosing specific tree genotypes with preferred mycobiome could lead to prolonged resistance against *H*. *parviporum* in diseased sites.

Norway spruce is an essential tree species for ecological and economic reasons. The mycobiome associated with Norway spruce includes various groups of fungi, such as mutualistic, saprotrophic, and pathogenic fungi. The composition of these fungal communities can vary depending on factors such as soil properties [26], tree genotypes [21, 27, 28] and environmental conditions [20]. Mycorrhizal fungi form a well-known mutualistic symbiotic relationship with the tree host. Part of trees’ mycobiome are also fungal endophytes that live non-symptomatically within plant tissues throughout their whole life cycle or at substantial period of time without inflicting any visible detrimental effects to the host [29, 30]. The extensive range of fungal endophytes found within individual hosts has prompted a surge in research focused on investigating the beneficial roles of fungal endophytes in forest trees [13].

In Norway spruce roots, *Phialocephala fortinii* – *Acephala applanata* species complex (PAC), which are dark septate endophytes (DSE), are the most abundant fungal endophytes [23, 31, 32]. DSE have high melanin concentrations and microsclerotia in their hyphae, which could be why they are able to offer protection to the host roots in extreme habitats [8, 33], as they are hydrophobic, drought-resistant, and capable of withstanding repeated freeze-thaw cycles [34].

Previous studies have demonstrated the increased positive effects of endophytes on the growth and protection against pathogens in forest trees [35–37]. One DSE, *Phialocephala sphareoides*, has been shown to enhance the Norway spruce root and seedling growth [24] and disturb the infection capability through roots of the pathogen in Norway spruce [37].

Trees are directly impacted by climatic extremes such as higher temperatures and drought, leading to water scarcity. These extremes indirectly render trees more vulnerable to pathogens [38, 39]. This affects host-microbiome (pathogens included) interactions, resulting in increased occurrence of disease outbreaks caused by previously harmless fungi [40]. The warming climate also allows fungi to emerge in more northern regions, resulting in unexpected new outbreaks [41, 42]. Overall, the distribution and evolvement of forest pests and pathogens are being changed as a result of climate change. There is particular concern about the effects of a warmer climate and drought on the interactions between plants and pathogens [43, 44].

Besides the increased interest in mycobiomes and their impact on tree health, understanding the relationship between plants and mycobiome remains challenging. It is hypothesized that the phloem endophytic mycobiome is mainly horizontally transmitted [45] and that the root mycobiome may change after seedlings are planted in field [46]. Overall, the fungal community associated with Norway spruce is complex and dynamic. Elucidating the specific fungal species and their interactions that contribute to the resistance of Norway spruce can deepen the understanding of the multiple interactions between plants and fungi. Further studies are required to fully understand mycobiome’s role in tree health and development. Taken together, the composition of the mycobiome is shaped by factors such as host plant genotype, environmental conditions, and interactions with other microorganisms. By studying how these factors contribute to the mycobiome’s makeup, we can better understand the intricate relationships between plants and their microbial communities. This knowledge can then be applied to develop more effective strategies to promote tree health and combat forest diseases.

To test the “mycobiome-associated-fitness” hypothesis, we compared control seedlings and seedlings artificially inoculated with *H. parviporum* for necrosis development and mycobiome composition under well-watered and drought-stressed conditions. Our aims were to determine the phloem and root tissue-specific mycobiomes and core species at Norway spruce genotype/family levels and the changes in mycobiome composition upon *H*. *parviporum* challenge and drought stress.

## Materials and methods

### Fungal and plant material

Fungal material consisting of two heterokaryotic strains of *H. parviporum* was obtained from the strain collection of Natural Resources Institute Finland. The strains, collected by Dr. Tuula Piri, include Hpa 1 (strain number: SB 2005 9.16), isolated from a Norway spruce stump in Solböle, Finland, and Hpa 2 (strain number: SB 2014 2.69), isolated from a Norway spruce seedling in Solböle, Finland [18]. Prior to the inoculations, the strains were cultured for 2 weeks at 21 °C, in darkness, on 1.5% Malt Extract Agar (MEA) in a growth chamber (Memmert HPP 750 constant climate chamber).

Three-year-old Norway spruce rooted cuttings used in this study originated from the Haapastensyrjä field unit (60°37′34.9“ N 24°27’34.9”E) of the Natural Resources Institute Finland (Luke). The rooted cuttings were initially grown outside in the field of Haapastensyrjä field unit before being collected and sent to Göttingen, Germany. On March 5, 2020, the plants were planted in 3-l plastic pots filled with 2.5 L of fertilized peat (Floragard, TKS®2 Instant Plus, PERLIGRAN® Extra 2–6 mm, Hermann Meyer KG, Rellingen, Germany) and placed in the greenhouse facilities at the University of Göttingen, Germany (51°33′28.4“ N 9°57’30.5”E). Plant material consisted of seven genotypes from four half-sib families: genotypes 38–3 and 38–8 (from family ID 38); genotype 40–41 (from family ID 40); genotypes 41–36 and 41–44 (from family ID 41); and genotypes 43–12 and 43–15 (from family ID 43) (Table 1).

**Table 1:** Family ID, genotype ID, average starting height, average diameter (measurement taken ~ 5 cm from stem base), number of cuttings in each genotype and treatments for Norway spruce. Hpa 1 presents the strain *H*. *parviporum* no 1, and Hpa 2 *H*. *parviporum* no 2. Control refers to mock control (Malt), and NT refers to non-treated

Family ID	Genotype ID	Average starting height (cm)	Average diameter (mm)	No. of cuttings per genotype	Watering treatment	Number of cuttings per inoculation
F38	38–3	44	7	10	Lower	Hpa1 (3), Hpa2 (3), Control (3), NT (1)
F38	38–8	37	7	10	Optimum	Hpa1 (3), Hpa2 (3), Control (3), NT (1)
F40	40-41O	40	6	3	Optimum	Non-treated
F40	40-41D	40	8	4	Lower	Non-treated
F41	41–36	44	7	10	Lower	Hpa1 (3), Hpa2 (3), Control (3), NT (1)
F41	41–44	42	7	10	Optimum	Hpa1 (3), Hpa2 (3), Control (3), NT (1)
F43	43–12	34	5	10	Lower	Hpa1 (3), Hpa2 (3), Control (3), NT (1)
F43	43–15	38	7	10	Optimum	Hpa1 (3), Hpa2 (3), Control (3), NT (1)

Half of the genotypes (38–8, 41–44 and 43–15) were optimally watered for 16 weeks, while the other half (genotypes 38–3, 41–36 and 43–12) were subjected to drought stress by receiving half of the optimal watering amount (details on the watering amount are described in [18]). The watering quantity varied based on the soil moisture content (see [18]), which was constantly measured using a tensiometer (Supplementary Fig. S1A). Moreover, 10 ramets per genotype were used as biological replicates for inoculation treatments: three replicates were inoculated with the *H*. *parviporum* strain 1 (Hpa 1), another three replicates with strain 2 (Hpa 2), and, finally, another three replicates were mock-inoculated with 1.5% Malt Extract Agar. The remaining ramet for each genotype was non-treated. Moreover, a seventh genotype (40–41, from family ID 40) was used as internal control, with three ramets being optimally watered and four subjected to drought stress, and no inoculation treatment was applied. The experiment was conducted under standard ambient lighting, and temperatures in the greenhouse averaged 31.8 °C in July, 25.9 °C in August, 23.2 °C in September, 15 °C in October, and 11.8 °C in November (Supplementary Fig. S1B).

### Inoculation and sampling

Inoculation was done by boring with a 5 mm cork borer through the bark of the seedlings (at the lower stem region) to reach the sapwood surface and placing equal-sized 1.5% MEA plugs of the inoculum (*H*. *parviporum*) on the exposed surface before wrapping it with parafilm to prevent falling off or drying out. The exact process was carried out also for the mock control with the inoculum replaced with sterile 1.5% MEA plugs. For each of the 67 trees, the phloem tissue was harvested in tubes, immediately frozen in liquid nitrogen, and transferred to − 80 °C for storage. The bark was scraped with a scalpel (after freezing) to measure the necrosis. The vertical and horizontal lesions in the phloem and sapwood were measured with a digital caliper.

Additionally, from the root tissues, twelve samples were collected from mock-inoculated control seedlings of families 38 and 41 and six from the non-treated seedlings (Family 40) (Table 1).

### DNA extraction, amplification and sequencing

The root samples were washed to remove the soil and other debris, the bark was removed from the stem, and the phloem was collected from and around the inoculation point. One hundred fifty (150) mg of the phloem or root material was ground into powder in liquid nitrogen with a sterile mortar and pestle. The genomic DNA (gDNA) was extracted using the modified cetyltrimethylammonium bromide (CTAB) method [47], and the gDNA concentration was measured using Qubit fluorimeter (Life Technologies) quantification. The PCR amplification of the internal transcribed spacer 2 (ITS2) region was performed with primer pair ITS3F and ITS4R. The amplicons were sequenced with the Illumina Sequencing platform to generate paired-end raw reads of 250 bp length. The PCR, library preparation and sequencing were carried out by Novogene (Cambridge Science Park, United Kingdom). The DNA amplicons were run on a 1.5% agarose gel at 60 V for 90 min.

### Raw data processing

Paired-end reads were allotted to the samples based on their unique barcodes and truncated by cutting off the barcode and primer sequences. The reads were merged using FLASH (V1.2.7) [48] and spliced where there was an overlap between the reads and those generated from the opposite ends of the same DNA fragment. The raw sequences pre-processed by Novogene with the barcodes and primers removed were used for further analysis. The bioinformatic platform Quantitative Insights into Microbial Ecology (QIIME2–2021.8) [49] was used to analyze the mycobiome composition and diversity associated with the samples. The raw sequences were imported into QIIME2 and processed using the DADA2 pipeline to denoise and infer the exact amplicon sequence variants (ASVs). To study the microbial community composition in each sample, the ASVs were aligned and annotated using the UNITE QIIME release for fungi (Version 10.05.2021) [50] database. Aligning sequences with 99% similarity were assigned to the same ASV. The sampling depth was normalized using the sample with the minimum sequencing depth, and the normalized sampling depth was sufficient enough for the subsequent analysis. Alpha rarefaction curves were generated using QIIME2 to assess sample coverage and whether the normalized sampling depth was enough to capture fungal diversity. The datasets produced and/or examined in the present research are accessible at NCBI under BioProject PRJNA990335; SRA number SUB13564759 (SRR25109452 - SRR25109534).

### Statistical analysis

Necrosis analysis was carried out for samples across families and genotypes. The data distribution was assessed employing the Shapiro–Wilk test [51]. The Bartlett test was employed to evaluate the homogeneity of variances for normally distributed data. Analysis of variance (ANOVA) analysis was carried out, followed by a Tukey HSD post hoc. Homogeneity of variance for not-normally distributed data was assessed using Levene’s test; due to the homoscedasticity of the data, a Kruskal-Wallis analysis was carried out.

Mycobiome analysis was carried out separately for phloem and root samples, and this was assessed against family/genotypes and water treatments. Analysis was performed, excluding ASV values from inoculated *Heterobasidion*. The normal distribution of our data was evaluated using the Shapiro-Wilks normality test [51]. To analyze the non-normally distributed data, the non-parametric Kruskal-Wallis test was employed to identify and compare the differences in diversity and prevalence among fungal communities with variables greater than two. Post hoc analysis was carried out using Dunn Bonferroni. The non-parametric Wilcoxon rank sum test was used for factors with two variables. The *p*-value adjustment method employed was Bonferroni. For normally distributed data, the standard t-test was used for factors with two variables, while the ANOVA was used for factors with more than two variables. This was followed by a Tukey HSD when factors significantly varied.

Alpha diversity was assessed using the observed features/ASVs, Simpson and Shannon-Wiener diversity indices with the R package vegan [52, 53], and using the adonis function to perform a permutational analysis of variance (PERMANOVA) based on Bray-Curtis with 999 permutations. Bonferroni post hoc analysis was done when the fungal taxa significantly differed. Principal coordinate analysis (PCoA) was used to visualize the fungal community structure. This was performed using vegan [52] and ggplot2 [54] packages in R. A correlation analysis was carried out between the necrosis and growth variables and the most abundant identified fungal genera. All reads for each genus were combined, and the 50 most abundant identified genera were used for the correlation using Spearman’s rank correlation. The taxonomic classification / fungal diversity for the top 50 species was visualized in a tree format using the heat tree function in the Metacoder package in R [55]. Functional annotation of the resulting fungal communities in roots was assessed using the FungalTrait database [56]. We analyzed the stability between dark septate endophytes (DSEs) and ectomycorrhiza fungi (ECM) between water treatments using the frequency of the major DSEs and ECMs present in our samples. Indicator species for each family were identified with the R package indicspecies [57]. A *p*-value below 0.05 was considered statistically significant in this study.

### Correlation between genetic distance and distance among taxa

Using genomic information of the seven genotypes used in this study (data not shown; Chano et al., in preparation), we performed a Mantel test [58] to infer the correlation between pairwise genetic distance and the calculated pairwise distances from taxa abundance. As the genotypes were split into two sets subjected to different water regimes (genotypes 38–8, 40–41, 41–44 and 43–15 for optimum watering and genotypes 38–3, 40–41, 41–36 and 43–12 for drought condition), two separate Mantel tests were performed to avoid the effect due to watering. In addition, just three of the four genotypes from each set were used for inoculation experiments, so just untreated plants were considered. Distance matrices were generated with the function dist() in R [53] and Mantel test was performed by using the function mantel.rtest() from the R package ade4 [59], including 9999 permutations.

### Fungal isolation, identification, and dual-culture testing

Based on data analysis, fungal endophytes were recovered from the three Norway spruce families (38, 41, 43). The soil was removed from the roots under running tap water. The roots were cut into small pieces (1 cm) and surface sterilized (70% ethanol 1 min, 2% sodium hypochlorite (NaOCl) 30 sec, rinsed in sterile ddH_2_O). The surface-sterilized roots were plated on 1.5% malt extract (MEA). The plates were stored in darkness at 19 °C with 75% relative humidity. Based on morphology, we chose one endophyte from each family for dual-culture-inoculation against *H. parviporum*. The DNA was extracted as described in [60], and species of endophytes were confirmed with ITS regions with primer pair ITS1-F [61] and ITS4 [62]. Purified PCR products were sequenced using the ITS4 at Microsynth SEQLAB (Germany).

The chosen three endophytes and *H. parviporum* were plated on a 1.5% MEA at 0.5 cm from the edge of 8.5 cm Petri plates for a dual culture antagonisms assay (paired growth assay). The growth of *H. parviporum* was measured on days 7 and 10. The sequences for the isolates are available under the ascension numbers: OR167041, OR167042, and OR167043.

## Results

After denoising and quality filtering of the phloem samples, 7629 features/ASVs were obtained with a total frequency of 7,973,147. Frequency per sample ranged from 64,507 to 167,468. Normalization was carried out using the sample with the minimum sampling depth (64,507) to include all our samples in the analysis - such that all samples have the same sequencing depth. The 7629 features/ amplicon sequence variants (ASVs) were obtained from phloem samples and clustered into 1181 identified ASVs. From root materials, two samples were excluded due to bad quality. From the remaining 16 samples, 1793 features/ASVs were obtained with a total frequency of 2,290,039. Frequency per sample ranged from 109,538 to 165,187. Sample normalization was done with a minimum sampling depth of 109,538. The total of 1793 features obtained was clustered into 454 identified ASVs.

The clustered ASVs were used for further analysis. The data were explored to see whether specific taxa differed between the genotypes and water treatment.

The soil moisture content differed significantly between the treatments from the fifth week (*p* = 2.79e− 06, Supplementary Fig. S1A). In phloem, the highest percentage of the phloem taxa belongs to the Ascomycota phylum with 72 % (72%), followed by Basidiomycota with 24 (24%) percent. Mortierellomycota had 2 %, while Chytridiomycota and Mucoromycota each had 1% of the total fungal composition. Other negligible taxa include Aphelidiomycota, Basidiobolomycota, Glomeromycota, and Rozellomycota. In roots, Ascomycota also occupied the largest percentage of the mycobiome (75%), followed by Basidiomycota (18%). The other taxa included Mortierellomycota (3%), Mucoromycota (2%) and Chytridiomycota (1%). Other negligible root taxa include Aphelidiomycota, Basidiobolomycota, Glomeromycota, and Rozellomycota. A comparison of the most abundant fungi in the phloem and the root shows some uniqueness and overlap, as some fungi are found both in the stems and roots but with differences in their abundances/expression levels (Table 2).

**Table 2:** Top 12 identified Amplicon Sequence Variants in phloem and root tissues

ASV_ID	Phylum	Species	Total reads	Relative abundance (%)
Phloem				
ASV_1	Ascomycota	*Paraphaeosphaeria neglecta*	1,584,628	19.9
ASV_2	Ascomycota	*Setomelanomma holmii*	1,466,026	18.4
ASV_3	Ascomycota	*Lachnum virgineum*	626,774	7.9
ASV_4	Ascomycota	*Angustimassarina acerina*	493,135	6.2
ASV_5	Ascomycota	*Phialocephala fortinii*	272,659	3.4
ASV_6	Ascomycota	*Lachnum* sp.	216,555	2.7
ASV_7	Basidiomycota	*Heterobasidion parviporum*	208,438	2.6
ASV_8	Ascomycota	*Phialocephala* sp.	168,709	2.1
ASV_9	Ascomycota	*Brunnipila fuscescens*	98,498	1.2
ASV_10	Ascomycota	*Cadophora* sp.	78,448	1.0
ASV_11	Basidiomycota	*Amphinema* sp.	76,332	1.0
ASV_12	Ascomycota	*Xenochalara* sp.	44,594	0.6
Roots				
ASV_1	Basidiomycota	*Amphinema* sp.	312,469	13.6
ASV_2	Ascomycota	*Trichophaea* sp.	282,120	12.3
ASV_3	Basidiomycota	*Amphinema byssoides*	253,470	11.1
ASV_4	Ascomycota	*Phialocephala fortinii*	224,494	9.8
ASV_5	Basidiomycota	*Thelephora terrestris*	222,772	9.7
ASV_6	Ascomycota	*Wilcoxina* sp.	174,489	7.6
ASV_7	Ascomycota	*Hyaloscypha finlandica*	92,689	4.0
ASV_8	Ascomycota	*Dactylonectria macrodidyma*	65,356	2.9
ASV_9	Ascomycota	*Dactylonectria anthuriicola*	58,814	2.6
ASV_10	Ascomycota	*Ilyonectria mors*-*panacis*	30,738	1.3
ASV_11	Ascomycota	*Hyaloscypha_variabilis*	24,039	1.0
ASV_12	Ascomycota	*Lecanicillium_primulinum*	16,814	0.7

**Fig. 1:**
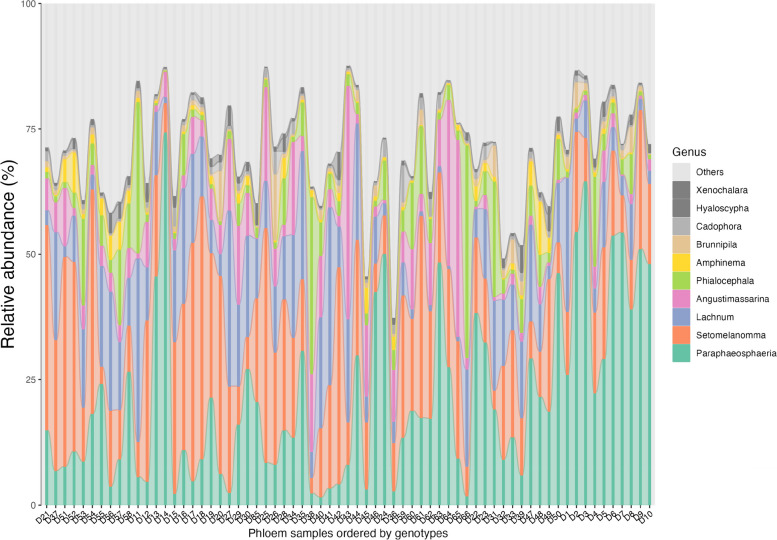
The relative abundance of the top 10 taxa of the 67 phloem samples from different genotypes labelled D1-D67, ordered according to genotypes (38–3, 38–8, 40-41D, 40-41O, 41–36, 41–44, 43–12, 43–15. D1-D67; Supplementary table S1)

**Fig. 2:**
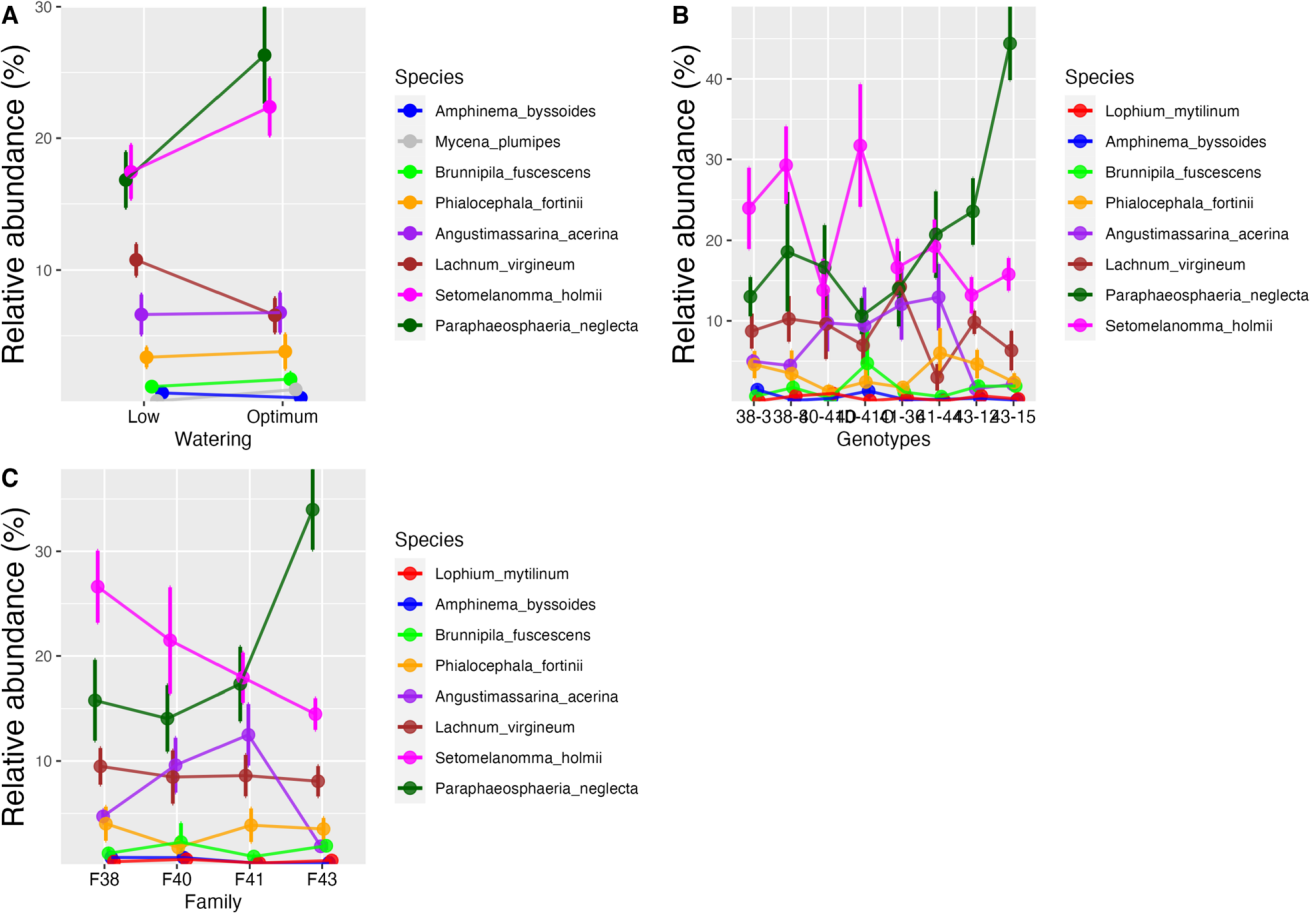
Relative abundance differences of the top eight taxa in the phloem along **A)** water treatment, **B)** genotypes, **C)** family. 40-41D are with low-water treated genotypes in family 40, while 40-41O are optimally watered (Connecting lines are included to visualize common patterns)

There were variations among relative proportions of taxa across all samples; certain species or genera were more abundant in some genotypes and treatments than others. Overall, *Amphinema* sp. were the most abundant in roots (Table 2). *Paraphaeosphaeria neglecta,* followed by *Setomelanomma holmii,* were the most abundant fungi in phloem samples (Table 2, Fig. 1). *Paraphaeosphaeria* and *Setomelanomma* genera displayed a similar pattern in their response to water treatment in the phloem. There was a higher abundance in plants that were optimally watered than in plants with low water availability (Fig. 2A). This pattern was consistent across the different families and genotypes, except for family 40, where *Setomelanomma holmii* showed the opposite trend between the different water treatments, with higher presence in low-watered plants (Fig. 2B). Genotype 40–41 (low-watered) had a higher presence of *Paraphaeosphaeria neglecta* than the optimally watered plants with the same genotype (Fig. 2B). *Phialocephala fortinii* has the lowest abundance in family 40, which was non-treated (Fig. 2C).

### Results for phloem mycobiome

#### Genotype variation

**Fig. 3:**
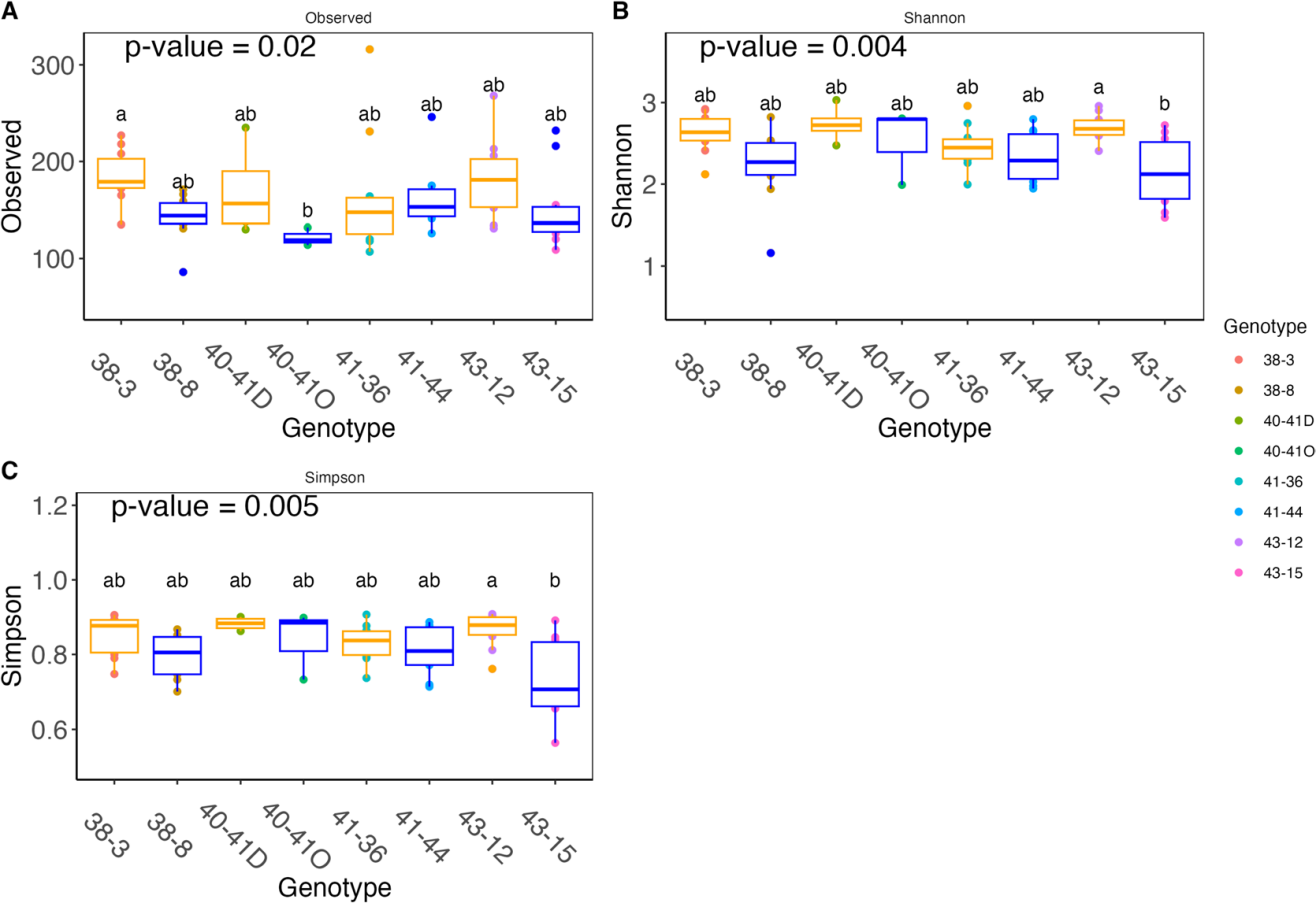
Alpha diversity among phloem genotypes. **A** observed amplicon sequence variants, **B** Shannon diversity index, **C** Simpson index. Different letters above plots denote significantly different groups after the post hoc test. Low-watered genotypes (orange colour) are 38–3, 40-41D, 41–36 and 43–12. Optimally watered plants (blue colour) are 38–8, 40-41O, 41–44, and 43–15

Alpha diversity differed significantly (*p* < 0.05) among genotypes in phloem samples (Fig. 3). There were significant differences in the alpha diversity among genotypes based on Kruskal-Wallis tests, observed ASVs (*p* = 0.02), Shannon (*p* = 0.004) and Simpson indices (*p* = 0.005) (Fig. 3). Based on observed ASVs, there were significant differences between genotypes 40-41O and 38–3 (*p* = 0.02) (Fig. 3A). Simpsons and Shannon’s diversity indices showed significant differences only between genotypes 43–12 and 43–15 (*p* = 0.02) (Fig. 3B, C).

**Fig. 4:**
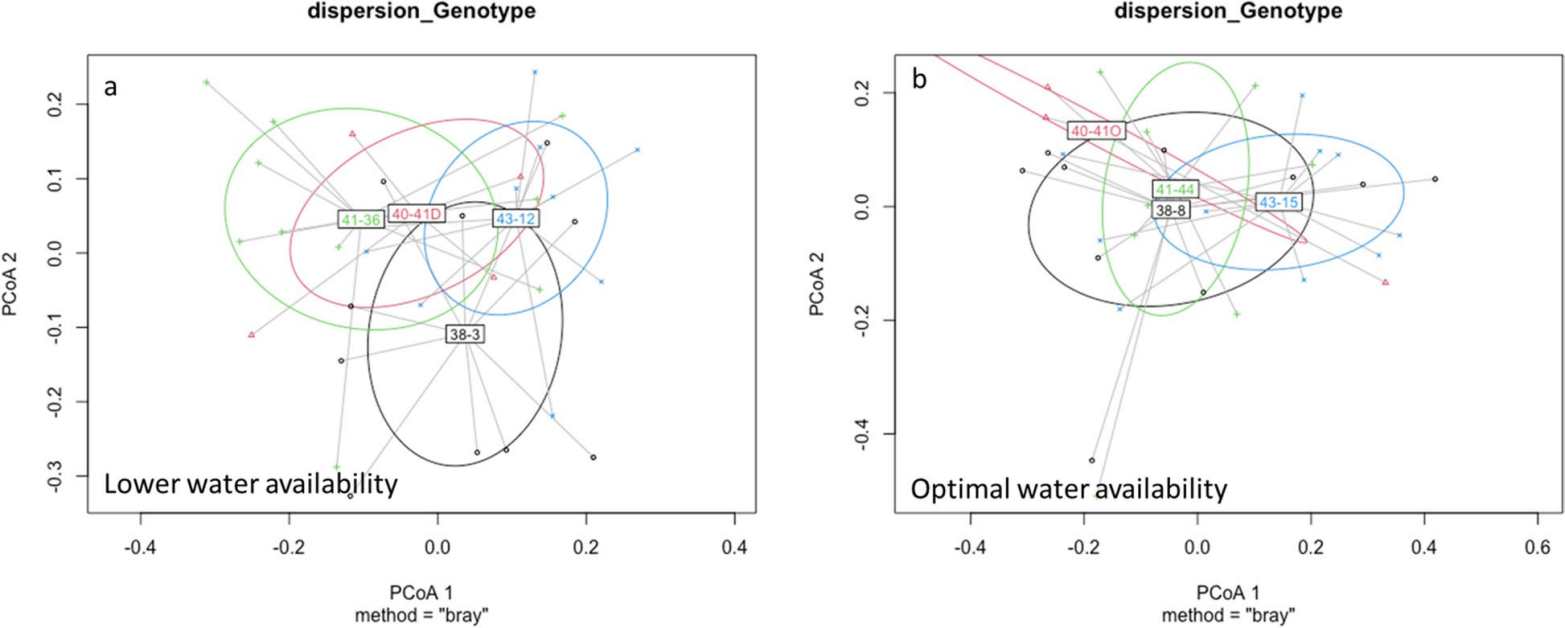
Dispersion of fungal communities among genotypes based on Bray-Curtis **A)** low-water treated plants **B)** optimally watered

Results from the PERMANOVA analysis showed significant differences (R^2^ = 0.23, F = 2.52, *p* = 0.001) among the genotypes in the phloem mycobiome (Supplementary Fig. S2). To test the impact of the water treatment, we compared the dispersion of fungal communities (dispersion genotypes) inside the corresponding group. Based on the permutational analysis of variance (PERMANOVA) of dispersion genotypes for each water treatment, meaningful differences were found between dispersion genotypes for lower water conditions (*p* = 0.001), but not for the optimally watered plants (*p* = 0.07) (Fig. 4). Genotype 38–3 seems located further away from the other genotypes (Fig. 4a), and genotypes 41–36 and 43–12 also appeared to cluster further apart from each other.

The results of the taxonomic classification of the phloem mycobiome for genotypes were visualized using the heat tree matrix (Supplementary Fig. S3). Taxa are represented as nodes, and sizes and colours indicate their abundance. Each taxon is coloured by the log-2 ratio of the median phloem ASV counts observed at each genotype. The colouring also shows significant differences between the median proportions of ASV counts for samples from the different genotypes. The colour intensity is proportional to the log2 ratio of the differences. For instance, differentially expressed taxa were different between water treatments between genotypes of family 43. There were more abundant species specific to Genotype 43–12 than 43–15. Family *Venturiaceae* and *Paraphaeosphaeria neglecta* were more abundant in genotype 43–15, while some of the taxa more abundant in genotype 43–12 included *Amphinema* sp., *Thelephora terrestris*, *Mycena plunipes, Neonectria tsugae* and *Spirophaera floriformis* (Supplementary Fig. S3).

#### Water treatments

**Fig. 5:**
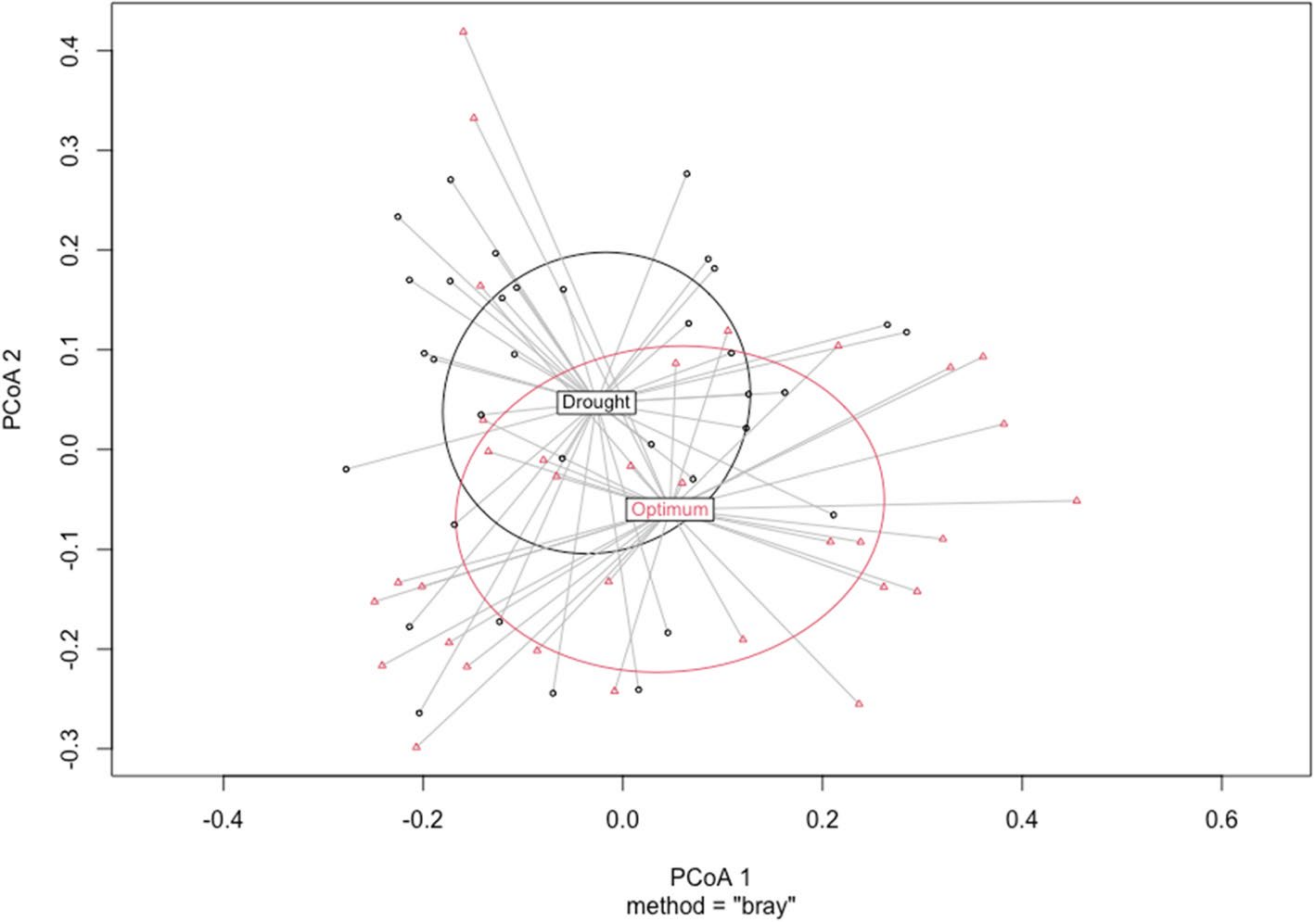
Fungal communities’ dispersion among watering treatments based on Bray-Curtis distances

Alpha diversity also differed significantly between watering treatments across all indices in the phloem mycobiome (Supplementary Fig. S4). Observed features had a *p-*value of = 0.007, while both Shannon and Simpson indices had *p*-values of = 0.0002 (Supplementary Fig. S4). There was higher fungal diversity in the drought-treated plants than in the optimally watered plants. The permutation analysis of variance among the phloem mycobiome also showed slightly significant differences (R^2^ = 0.04, F = 2.60, *p* = 0.01) among the watering treatments (Fig. 5).

**Fig. 6:**
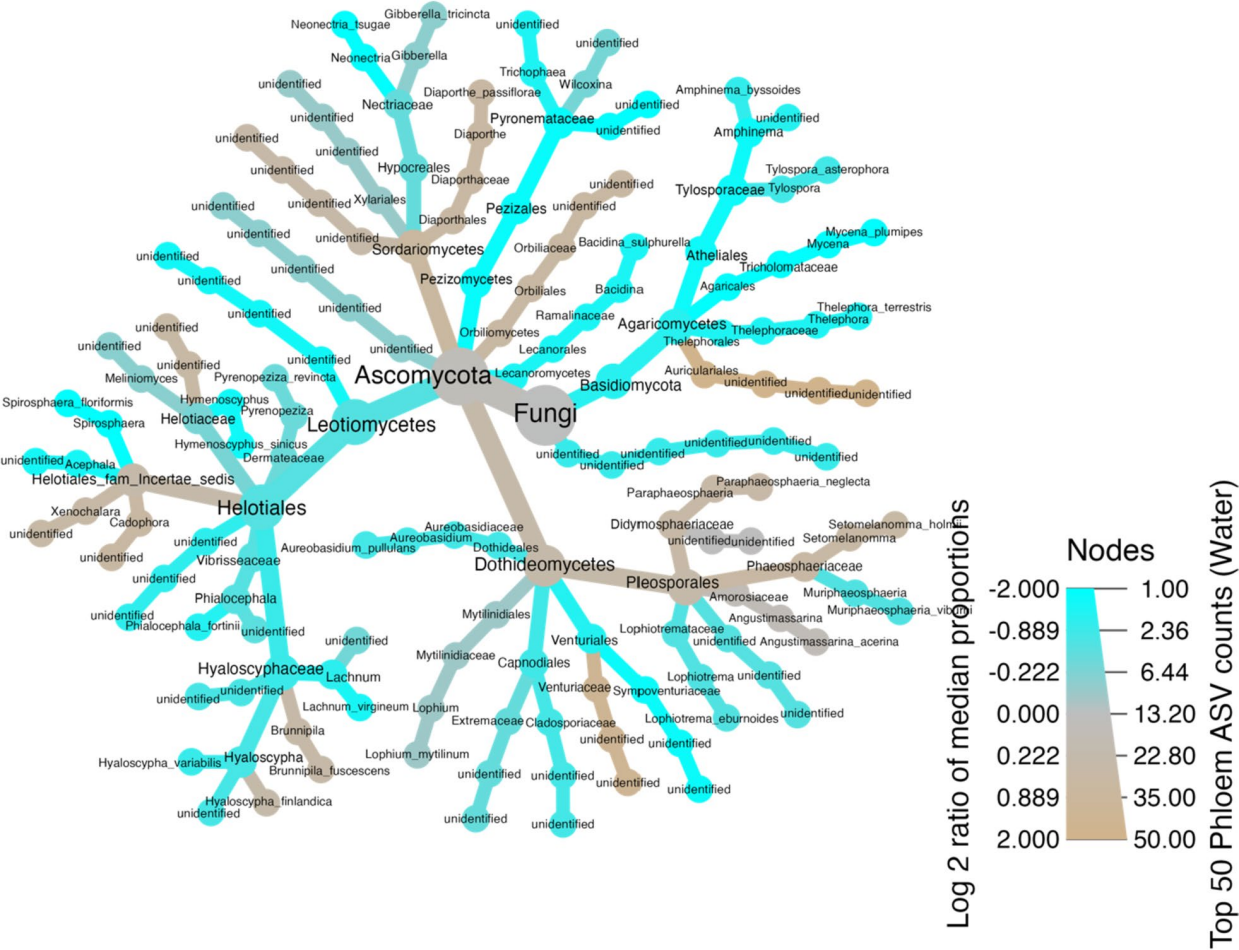
Phloem mycobiome abundance according to water treatment groups. The colour of each taxon represents the log-2 ratio of median proportions of phloem ASV counts observed at each water treatment category. Taxa-coloured tan have a higher relative abundance in the optimal watering category, while taxa-coloured cyan have a higher relative abundance in low-water treated plants

Using the heat tree function in the Metacoder package, the taxonomic classification of the phloem mycobiome (water treatment) was visualized (Fig. 6). The taxa are also represented here as the nodes, and the sizes and colours represent the abundances associated with the taxa. Taxa-coloured tan are more abundant in optimally watered plants, while those coloured cyan were more abundant in low-watered plants, although the significance level is not considered here. Low-watered plants are generally associated with more taxa than in the optimally watered plants. They contain genera such as *Phialocephala*, *Thelephora*, *Mycena*, *Tylospora*, *Amphinema*, *Neonectria*, and *Hyaloscypha*. The largest genera in the optimally watered group include; *Paraphaeosphaeria*, *Setomelanomma*, *Diaporthe*, *Xenochalara*, and *Brunnipila* (Fig. 6).

### Results for roots

#### Roots (genotype and water treatment)

No significant differences were observed in the mycobiome alpha diversity with the indices used (Shannon, Simpson and observed ASVs) for any factors (water treatment, family and genotypes). Also, no significant differences were observed in the permutation analysis of variance among the root mycobiomes (Supplementary Fig. S5). Taxa-coloured including *Thelephora*, *Trichophaea*, *Cadophora*, and *Angustimassarina* genera showed higher relative abundance in well-watered plants, while taxa such as *Amphinema*, *Phialocephala*, and *Mortierella,* among others, showed higher relative abundance in the drought-treated plants (Supplementary Fig. S5). Major dark septate endophytes (DSEs) genera in our samples included *Acephala*, *Cadophora*, *Cladophialophora*, *Exophiala*, *Gyoerffyella*, and *Phialocephala*. According to the FungalTraits database used for this study, the *Cadophora* genus was not stated to have DSE capabilities. We included it because it was identified based on other studies as a DSE. Ectomycorrhizal (ECM) genera included *Amphinema*, *Inocybe*, *Thelephora*, *Trichophaea*, *Tylospora*, and *Wilcoxina* (Supplementary table S2).

**Fig. 7:**
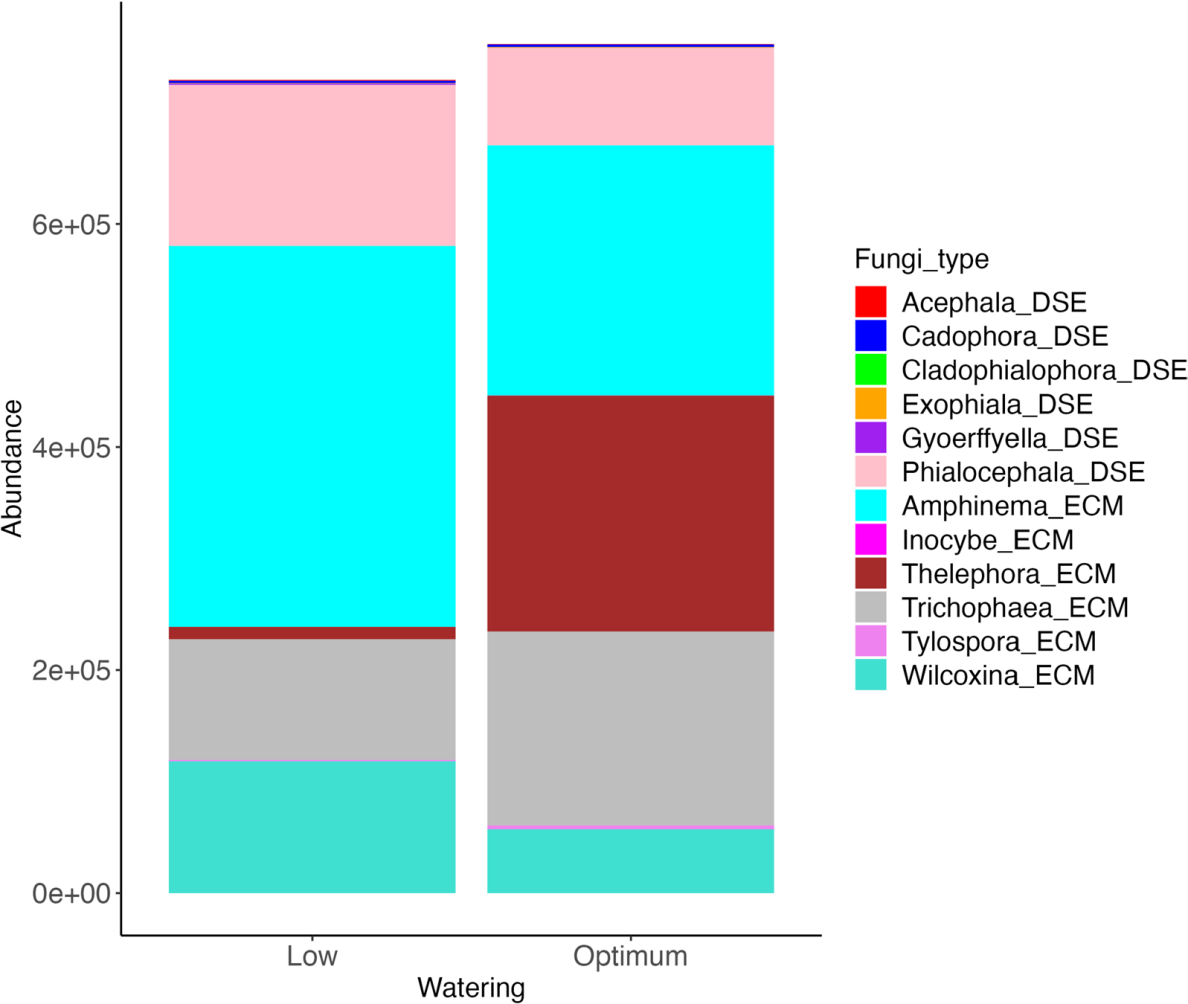
Relative abundances of dark septate endophytes (DSE) and ectomycorrhiza fungi (ECM) in the Norway spruce root tissues under different watering groups

ECMs and DSEs frequency varied between water treatments. The *Amphinema* genus was the most abundant among ECM fungi, followed by *Thelephora* and *Trichophaea*. *Thelephora*, *Trichophaea* and *Tylospora* seemed more unstable in low water conditions as their quantity was reduced (Fig. 7, Supplementary table S2). Under the low water treatment, the genera *Amphinema* and *Thelephora* had the highest presence, with 341,543 and 11,165 sequences, respectively. In contrast, these genera were less abundant under the optimum water treatment, with 224,396 and 211,607 sequences, respectively. The genera *Trichophaea* and *Phialocephala* also exhibited higher abundances under the low water treatment, with 108,305 and 144,730 sequences, respectively, compared to the optimum water treatment, where they had 173,817 and 87,491 sequences, respectively (Supplementary table S2). For the DSEs, the *Phialocephala* genus was the most abundant and more stable than most ECM fungi in low-water conditions. Other genera that exhibited higher abundances under the low water treatment include *Wilcoxina*, *Tylospora*, and *Cadophora*. In contrast, genera such as *Amanita*, *Capronia*, and *Phialophora* were not detected in the optimum water treatment, while they had low prevalence under the low water treatment.

### Interactions of phloem mycobiome with *H. Parviporum*

**Fig. 8:**
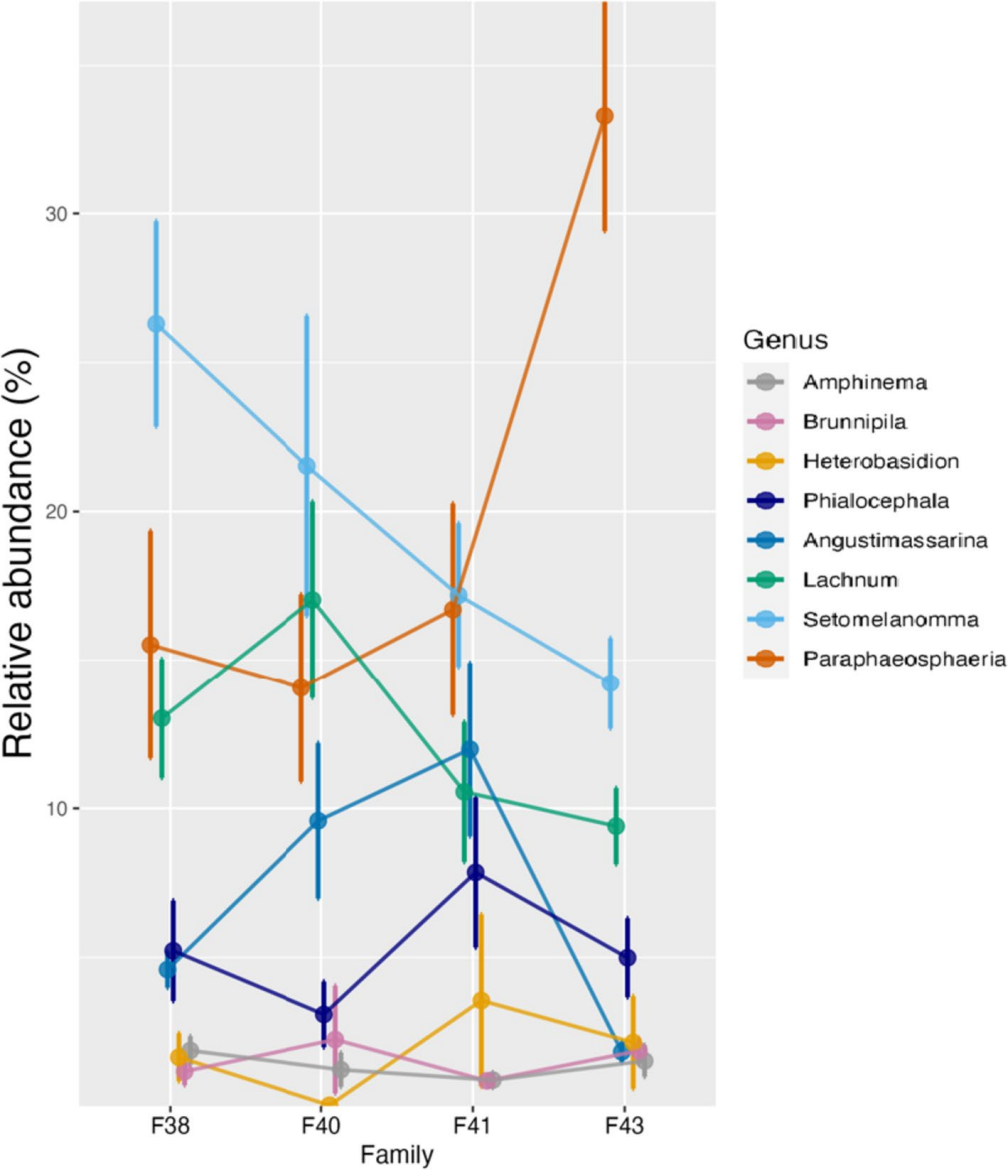
Relative abundance of taxa of the top eight fungi (phloem mycobiome) among families in the presence of *H*. *parviporum*. Connecting lines are included to visualize common patterns

The top eight genera in the phloem showed different patterns in their relative abundance across families (Fig. 8). *Paraphaeosphaeria* was present in similar quantities in families 38, 40 and 41, with lower abundance than in family 43. Among the top eight taxa, there were only significant differences between *Paraphaosphaeria* and *Angustimassarina* (Fig. 8). Family 43 showed a significantly higher relative abundance of the *Paraphaosphaeria* genus than families 38 and 41 (*p* < 0.01), while the relative abundance of the *Angustimassarina* genus differed significantly between families 43 and families 38 (*p* < 0.03) and 41 (*p* < 0.0002). *Setomelanomma* showed a decreasing trend from family 38 to family 43 (Fig. 8). More striking is the relationship between the inoculated *Heterobasidion* and DSE *Phialocephala,* which followed the same pattern. *Phialocephala fortinii* seemed to react to the presence of *H. parviporum*, as its abundance increased in concert with *Heterobasidion* (Supplementary Fig. S6).

**Fig. 9:**
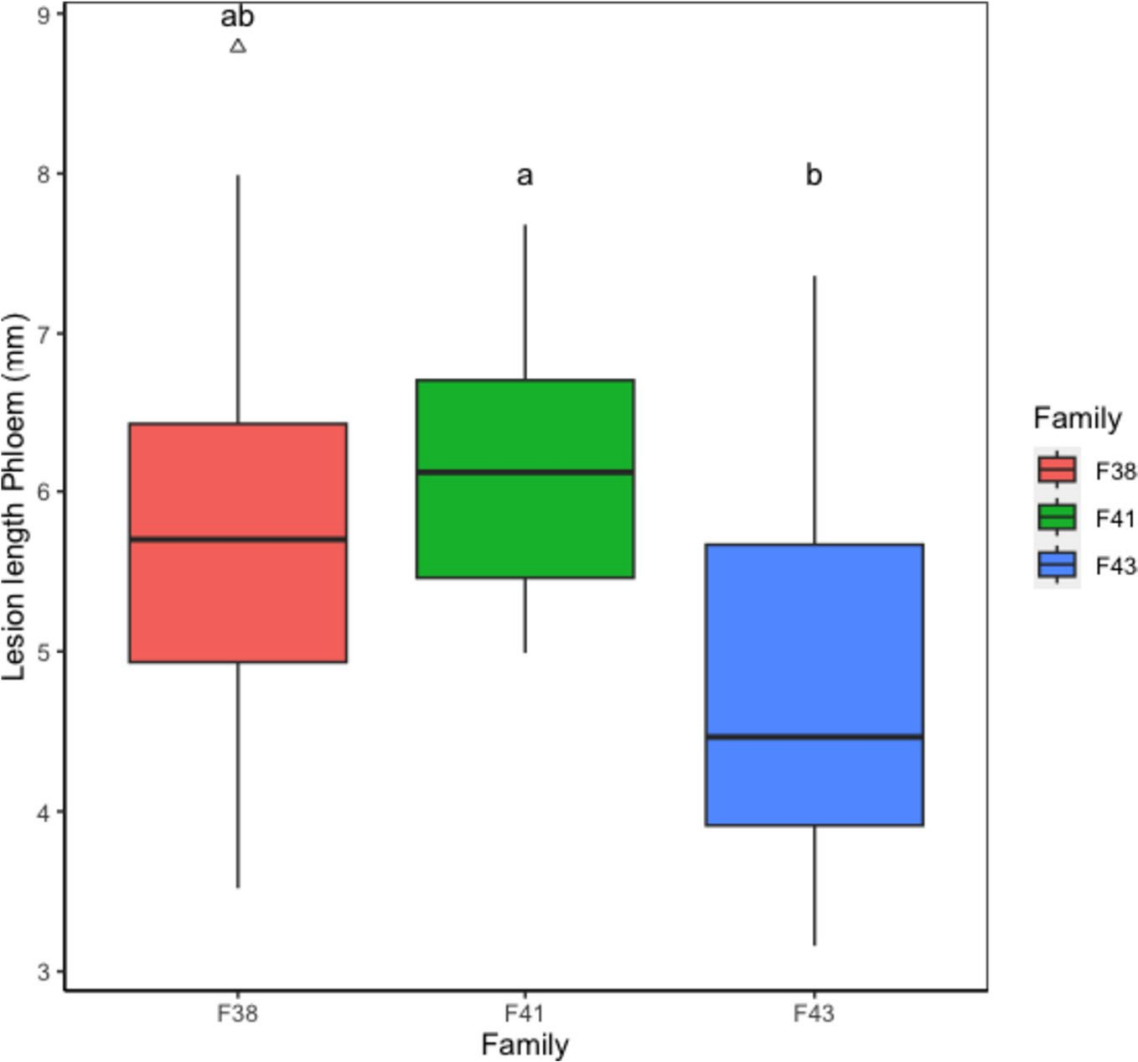
The effect of families on the lesion length in the phloem. Different letters above plots denote significantly different groups after post hoc test

There were no significant differences observed between the lesion length in sapwood and lesion width in both phloem and sapwood. The only significant differences observed among families were in the phloem lesion length (*p = 0.*04, Fig. 9). Family 41 has the highest lesion length but not significantly different from family 38. Family 43 has the lowest lesion length, significantly different from family 41 (Fig. 9). Family 41 has the highest amount of *Phialocephala fortinii* (Fig. 8), and also the highest lesion length (Fig. 9). Family 43 had the lowest lesion length compared to other families. Family 43 had 10 indicator species in the phloem (*Paraphaeosphaeria neglecta*, *Myxocephala albida*, *Metapochonia suchlasporia*, *Solicoccozyma terrea*, *Coniothyrium aleuritis*, *Mariannaea punicea*, *Mortierella cystojenkinii*) and three unidentified fungi.

**Fig. 10:**
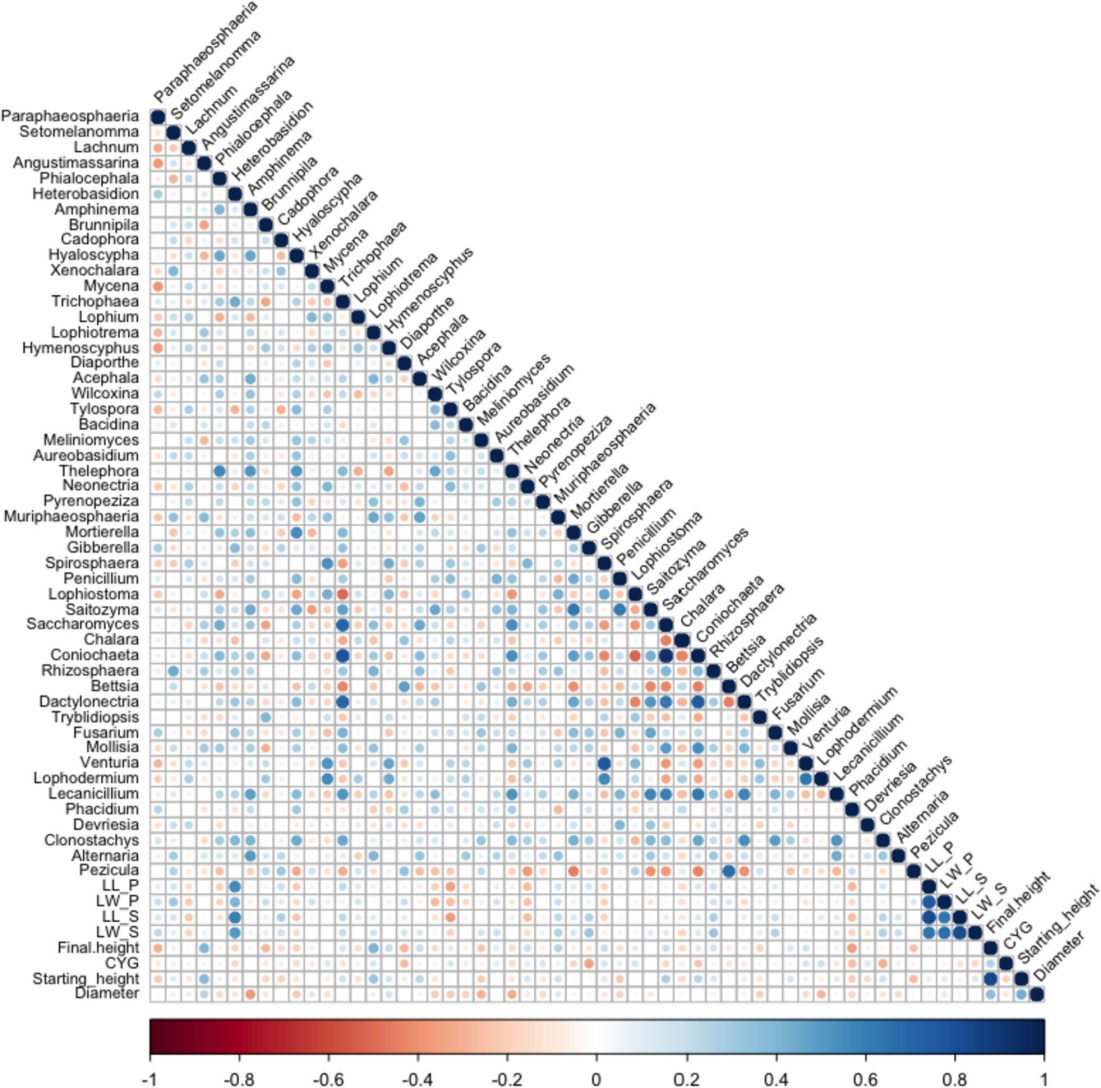
Correlogram representing the matrices of Spearman’s rank order correlation coefficient between the 50 most abundant identified different fungal communities and the Lesion length in the phloem (LL_P) and the sapwood (LL_S), Lesion width in the phloem (LW_P) and the sapwood (LW_S), final height of seedlings, current year growth (CYG), starting height and diameter measurements at the genus level. Positive (blue) and negative (red) correlations are only shown in the graph (*p* < 0.05)

The correlation analysis was conducted between the measured parameters (growth and lesion) and the top 50 fungal genera. There were strong positive correlations between the lesion length and lesion width in both phloem and sapwood (Fig. 10). The starting and final heights were also strongly positively correlated with each other. The diameter had moderate to strong negative correlations with almost all the fungal communities, e.g. *Amphinema*, *Wilcoxina*, *Tylospora*, *Thelephora*, and *Alternaria*. The starting height had weak to moderate negative correlations with *Paraphaeospharia*, *Meliniomyces*, *Penicillium*, *Phacidium*, *Devriesia*, and *Pezicula*. Positive weak to moderate correlations existed between starting height and *Angustinassarina*, *Lophiotrema*, *Acephala*, *Saccharomyces*, and *Coniochaeta*. *Phialocephala* genus had a weak negative correlation with the lesion measurements (lesion length in phloem and sapwood and lesion width in phloem) except lesion width in the sapwood. As expected, there were moderate to strong positive correlations between *Heterobasidion* abundance and lesion length in phloem and sapwood (Fig. 10). *Heterobasidion* abundance had a weak negative correlation to the growth variables. It was weakly to moderately negatively correlated to *Tylospora*, *Spirosphaera*, and *Chalara* genera. *Heterobasidion* was moderately to strongly positively correlated with fungi such as *Paraphaeosphaeria*, *Trichophaea*, *Mortierella*, *Gibberella*, *Fusarium* and *Coniochaeta* (Fig. 10).

### Isolated root endophytes and their interaction with *H. Parviporum*

**Fig. 11:**
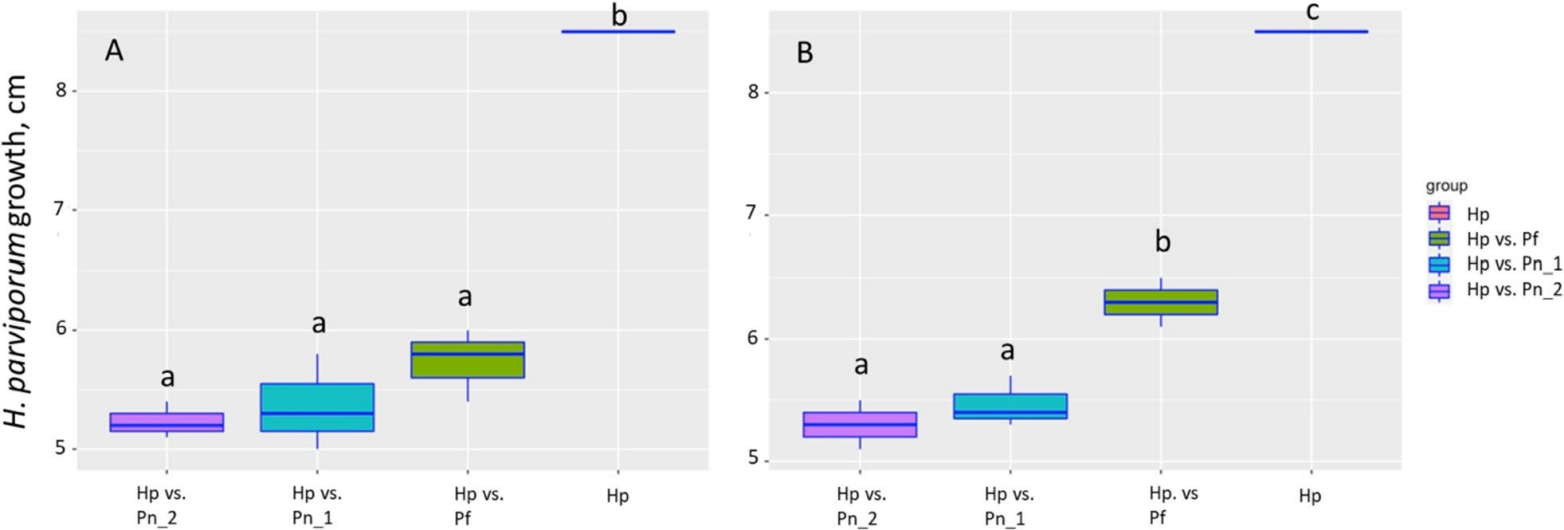
The growth of *H. parviporum* in dual-culture with *Paraphaeosphaeria neglecta* strain 1 (Pn_1) and 2 (Pn_2) and *Phialocephala fortinii* (Pf) after 7 days (A) and 10 days (B) *H*. *parviporum* (Hp) growing alone has reached the end of a Petri dish in 7 days (8.5 cm)

Two isolates were identified as *Paraphaeosphaeria neglecta* and one as *Phialocephala fortinii* (*Phialocephala fortinii*-*Acephala applanata* species complex). All three fungi inhibited the growth of *H. parviporum* after seven and 10 days (Fig. 11). However, the 2 *P. neglecta* strains were able to stop the growth of *H. parviporum* (Fig. 11B) as it could not grow further after 7 days. Endophytes can restrict the pathogen growth after 7 days (Fig. 11A), and *P. neglecta* strains have stronger inhibition compared to *P*. *fortinii* after 10 days.

### Correlation between genetic and mycobiome distances

No correlation was found in Mantel tests of pairwise comparisons of genetic distance and distances calculated from taxa abundance using genotypes used in both optimum watering and low-watered plants (simulated *p*-values of 0.5027 and 0.2538, respectively, based on 9999 permutations; Supplementary table S3).

## Discussion

Understanding the basis of plant fitness is vital for developing sustainable management strategies for forest ecosystems. Competitive fungal strains in the same niche would establish a new approach to forest disease research (or a novel paradigm). They could also form a basis for applied research using these principles to control forest and other plant pathogens. To get more insights into the possibility that the mycobiome is linked to increased tree resilience (“mycobiome-associated-fitness”), we monitored the mycobiome associated with different genotypes of Norway spruce under different watering conditions. To test the “mycobiome-associated-fitness” hypothesis, we also analyzed the interaction of artificially inoculated *H. parviporum* and the mycobiome. Our aims were to: 1) identify the specific mycobiome and core fungi within Norway spruce genotype/family level and changes in their relative abundance in relation to that of *H*. *parviporum* and 2) assess mycobiome stability under abiotic disturbance (lower water availability).

### Family and genotype impact on the mycobiome

We focused on the mycobiome within the stems and roots of Norway spruce. We observed that the mycobiome diversity was higher in stems than in roots. This is consistent with results on Norway spruce by [63], whose study also reveals that roots had the lowest diversity among all the tissue types tested. Some of the fungal genera found in shoots and roots of the study by [63] were also found in ours and include *Amphinema*, *Cadophora*, *Inocybe*, *Phialocephala*, and *Tylospora*. Several research studies have specifically focused on analyzing the mycobiome of needles in coniferous trees, particularly Norway spruce and pine species [21, 64, 65] and limited research on the phloem/stem mycobiome was conducted. Fungal species associated with Norway spruce needles include *Phoma herbarum*, *Alternaria alternata*, *Aureobasidium pullulans*, *Phialophora sessilis*, *Setomelanomma holmii*, *Sydowia polyspora*, *Aureobasidium pullulans*, *Cladosporium cladosporioides*, and *Rhizosphaera kalkhoffii* [21, 66, 67]. It has been shown that foliar endophyte communities strongly depend on the plant species they inhabit [68–71]. The needle [21] and bud [28] mycobiomes in Norway spruce vary largely in Norway spruce.

In our study, the diversity of the phloem mycobiome was found to differ significantly among the different genotypes of Norway spruce. Our genetic distance analysis shows no correlation between the genotypes and taxa communities, indicating no significant relationship between the genetic differentiation of the spruce genotypes used in this study and the composition of their fungal communities. The lack of correlation suggests that the genetic differences in our spruce seedlings are not the major drivers of mycobiome diversity. Other factors, such as environmental conditions (soil composition, moisture levels, temperature, and local microclimate, e.g. mycobiome present in the geographic location/origin of the cuttings), may have a greater impact on fungal community composition than the trees’ genetic makeup. Another implication could be fungi’s high adaptability and plasticity [72, 73], allowing them to respond to changing environmental conditions and establish associations with various host genotypes. Overall, the lack of correlation between genetic distance and mycobiome-based distances in Norway spruce genotypes implies that the mycobiome is shaped by a complex interplay of environmental factors and fungal adaptability, highlighting the need for further research to unravel the specific mechanisms driving these interactions. Multiple research studies have provided evidence indicating that the fungal communities are influenced by the genetic makeup of the host organism [19–21, 74–77]. A study by [65] demonstrated that the host genotype played a primary role in shaping the composition of the needle mycobiome in Norway spruce clones. It was observed that clones with a higher degree of genetic similarity exhibited a greater resemblance in their mycobiome profiles. In contrast, [64] could show no correlation between genotypic traits and mycobiome community in *Picea glauca*, but instead, the composition of the mycobiome showed a strong and positive correlation with the location of the trees that were sampled. Specifically, when two trees were close to each other, their needle mycobiome exhibited greater similarity.

Our study shows no genotype or drought effect on the root fungal communities, which is supported by a study from [78], which shows no variations in mycobiome across different populations of *Pinus pinaster*. In contrast, [79] revealed a host and drought effect on the Ectomycorrhizal fungi composition in *Pinus edulis*. In the course of our study, we encountered a fundamental limitation that warrants consideration. Specifically, our sample size comprised only seven genotypes from four families. This limited genetic diversity, while reflective of the available resources, may have constrained our ability to draw comprehensive conclusions, particularly in the context of our primary research aim. This limitation is further highlighted by the results of Mantel tests, which failed to reveal significant correlations between genetic distance and species abundance. While our findings provide valuable insights, it is essential to acknowledge the potential impact of this limitation. A larger and more diverse sample size could have offered a broader perspective on the relationships we explored. Further studies with a wider array of genotypes and families are needed to verify and expand on our findings.

### Mycobiome stability under abiotic disturbance (lower water availability)

In our study, most of the DSEs were more stable in their relative proportion in the low-watered plants than in the optimally watered plants as compared to the ECM fungi. *Thelephora terrestris* exhibited higher prevalence in our root samples but mainly in optimally watered plants. This fungus is one of the most common ectomycorrhizal fungi and has been suggested to be a strong competitor and capable of thriving in environments with limited species diversity [80]. The reduction in prevalence of ectomycorrhizal fungi in our study is consistent with results from [81], which also show that ectomycorrhizal fungi are more susceptible to drought than dark septate endophytes. Castaño et al. [78] could also show reductions in ECM fungi under drought stress. Drought associated with a warming climate will undoubtedly continue to impact (negatively) the future prevalence and functions of ectomycorrhizal fungi. Ectomycorrhiza (ECM) and dark septate endophytes (DSEs) are important fungi in plant growth and ecological functioning by forming mutualistic associations with tree roots, contributing to nutrient uptake and cycling [82] and helping to cope with stress [37, 83], and pathogen attack [84]. Changes in water availability, such as drought, can alter the abundance and functioning of fungal communities in ecosystems, which can, in turn, affect plant health, nutrient cycling, and ecosystem productivity [79, 85]. Based on the review by [86], drought has been suggested to lower mycorrhizal abundance. In a recent work by [76], members of the Ascomycota phylum were increased under flood conditions in roots of *Ulmus minor* genotypes resistant to Dutch elm disease (DED), while the presence of Basidiomycota members was reduced. However, the impact of drought caused the opposite effect, slightly increasing the presence of Basidiomycota members while Ascomycota decreased. Persistent low water conditions also limit ectomycorrhizal fungi abundance [87].

Interestingly, in our study the response of specific mycobiome genera to water availability differed across the genotypes and families (in the phloem mycobiome), indicating a complex interplay between genetic factors and environmental conditions. The results of the study suggest that the abundance of certain fungal genera in the phloem of Norway spruce trees is influenced by water availability. Generally, the genera *Paraphaeosphaeria* and *Setomelanomma* showed a higher abundance in plants that were optimally watered compared to plants with low water availability. This trend was consistent across most of the different families and genotypes studied, indicating a general response of these fungi to changes in water availability. But in contrast, the low-watered genotype of family 40 had a higher abundance of *Paraphaeosphaeria neglecta* than in optimally watered plants in the same genotype. This exception suggests that the relationship between fungal abundance and water availability may be more complex than a simple positive or negative correlation.

Water availability may be a driving factor in shaping the diversity and abundance of fungi within the phloem mycobiome. Water availability might also be triggering a reaction in the plant cells, causing shifts in the abundance of these species such that the low-watered plants are subjected to a more competitive environment and, therefore, are more likely to harbour diverse microbial communities that enable them to survive under such conditions. The effects of climate change with lower precipitation are shown to increase the frequency of drought and disease incidence rates [88]. Low water availability also alters the microbiome, particularly the bacterial composition in the soil [89]. Host mycobiome, depending on its composition and functional diversity among the co-inhabiting fungal species, can influence several processes responsible for plant growth [80]. These systems can, in turn, be affected by various factors, including environmental conditions and genetic variations within the host plant [70, 79].

### The mycobiome as antagonist towards pathogens and Heterobasidion

Our findings show varying degrees of necrosis and relative fungal abundances across different families. It is important to note that metabarcoding data provides only relative abundance of species. An increase or decrease in the relative abundance of a species between treatments does not necessarily imply a corresponding change in the biomass of the species, but can also be caused by variation in the prevalence of co-existing species. Family 43 had the lowest necrosis and stood out from the other families studied as having the highest number of indicator species in the phloem. This suggests that the phloem of trees in family 43 is particularly conducive to the growth of these fungi and that these fungi may have a closer relationship with these trees compared to the other families studied. Of particular note is *Paraphaeosphaeria neglecta*, which was found in similar quantities across families 38, 40, and 41 but was much more abundant in family 43. This indicates that this fungus may have a particularly close association with the trees in family 43. These results may have implications for understanding the interactions between fungal species and their impact on plant health in forest ecosystems. Fungi are known to enhance plant growth [37], protect and increase plants’ tolerance against stress, e.g. drought, high temperature, salinity, and pathogens [90–93]. The mycorrhizal fungus *Tricholoma vaccinum*’s protective function against pathogenic fungi was validated using dual cultures that included both *Botrytis cinerea* and *H. annosum* [84]. This experiment demonstrated decreased pathogen growth and increased survival rates of spruce trees. The symptoms on needles were also mitigated when the trees formed a symbiotic relationship with *T*. *vaccinum*.

Our antagonism assay shows *Phialocephala fortinii* and *Paraphaeosphaeria neglecta* to inhibit the growth of *H. parviporum*. Other species in the *Phialocephala* genus have been shown to inhibit the growth of *H*. *parviporum* in Norway spruce [24, 37]. Furthermore, our results also show that certain fungi, including *Setomelanomma holmii*, could significantly inhibit the growth of *Hymenoscyphus fraxineus* [94]. The function of *Setomelanomma holmii* in our study is, however, unknown. The ability of dark septate endophyte to suppress the growth of *H. annosum* was tested in a study by [95]; *Cadophora* sp. and *Phialophora mustea* were able to substantially reduce the growth of *H*. *annosum*. The inhibitory effect of fungal endophytes has also been reported with other tree species. A study by [96] could show several fungi with inhibitory potential against *Sphaeropsis sapinea*. Arnold et al. [97] also demonstrated the role of endophytes in plant defense in a tropical tree species. *Theobroma cacao* seedlings were pre-inoculated with endophytes before inoculating with *Phytophthora*. *T. cacao* leaves not treated with endophytes showed higher leaf area damage when inoculated with *Phytophthora* pathogen than leaves pre-inoculated with endophytes. This shows that the endophytes confer some form of protection against the pathogen, thus limiting pathogen infection. In *U. minor*, endophytes primed the plant immune system against the DED pathogen [98], and the inoculation with endophytes showing activity against DED promoted root growth and photosynthetic rates as well as resistance to the pathogen [99, 100]. Kosawang et al. [94] hypothesized that fungal endophytes associated with *Fraxinus* sp. could protect them from ash dieback and possibly act as biocontrol agents.

#### Higher fungal diversity in stressed plants

Our results reveal greater fungal diversity in plants subjected to drought stress than in optimally watered plants. The mycobiome’s response to drought can vary depending on the initial fungal diversity within the ecosystem. In some instances, environmental stress can reduce fungal diversity, particularly if sensitive species decline [101]. Conversely, it could favour either drought-tolerant fungal species that enhance the tree’s ability to cope with water stress by improving water and nutrient acquisition or benefit opportunistic pathogens that take advantage of the weakened host condition [101]. This diversification may be beneficial to the host, as in our case, there was an increased relative sequence abundance of fungal taxa with antagonistic effects on pathogenic fungi such as *Paraphaeosphaeria* and *Phialocephala* genera. These fungi were also shown to inhibit the growth of *Heterobasidion* in vitro in our study, thus they could potentially provide resistance against *Heterobasidion* infected trees in nature. However, it is worth noting that diversification may also have negative implications, such as the introduction of pathogenic fungi that could harm the tree host or compete with beneficial mycorrhizal fungi, reducing the tree’s access to water and nutrients [102].

## Conclusion

The results of this study highlight the importance of considering the genetic diversity of host plants when assessing the diversity and composition of fungal communities associated with them. *Paraphaeosphaeria neglecta* was an indicator species in family 43 that had the lowest necrosis, and it also seemed to interact with *H*. *parviporum*. Not much is known about this fungus, and we cannot fully ascertain whether it competes in the same niche or performs completely different functions. It was found as an endophyte, but could it also be an opportunistic saprophyte? Is it a beneficial or opportunistic fungus? The findings of this study shed light on the intricate relationships between fungal communities and water availability in Norway spruce genotypes. While the general trend is that fungal diversity decreases under water stress, the exceptions found in this study suggest that further research is needed to understand the factors influencing fungal communities in forest ecosystems fully.

These findings suggest that the phloem mycobiome of Norway spruce is shaped by a combination of genetic and environmental factors and that specific mycobiome genera may have adaptive responses to abiotic stress. Most ectomycorrhiza fungi were more susceptible to low water availability than dark septate endophytes. Few endophytes were able to restrict the pathogen growth. So, can “mycobiome-associated-fitness” be real? It is clear, however, that trees have both beneficial and opportunistic fungi. The mycobiome should be included in resistance studies and could be considered one factor in plants’ extended genotype variation against pathogens. How climate change will impact these fungi and their roles needs to be considered. Further research is required to explore the mechanisms underlying the observed differences in mycobiome diversity and composition among the genotypes and to determine the functional significance of these differences. These could have important implications for understanding the ecology and evolution of Norway spruce species and developing strategies for managing forest ecosystems under changing environmental conditions.

### Supplementary Information

**Additional file 1.**

## Data Availability

The datasets produced and/or examined in the present research are accessible at NCBI under BioProject PRJNA990335; ascension numbers SRR25109452 - SRR25109534. The sequences for the isolates are available under the ascension numbers OR167041, OR167042, and OR167043.

## References

[1] Yachi S, Loreau M (1999). Biodiversity and ecosystem productivity in a fluctuating environment: the insurance hypothesis. Proc Natl Acad Sci.

[2] Bengtsson J, Nilsson SG, Franc A, Menozzi P (2000). Biodiversity, disturbances, ecosystem function and management of European forests. For Ecol Manag.

[3] McCann KS (2000). The diversity–stability debate. Nature.

[4] Mitchell CE, Tilman D, Groth JV (2002). Effects of grassland plant species diversity, abundance, and composition on foliar fungal disease. Ecology.

[5] Pautasso M, Holdenrieder O, Stenlid J, Scherer-Lorenzen M, Körner C, Schulze E-D (2005). Susceptibility to fungal pathogens of forests differing in tree diversity. Forest diversity and function: temperate and boreal systems.

[6] Blumenstein K, Albrectsen BR, Martín JA, Hultberg M, Sieber TN, Helander M, Witzell J (2015). Nutritional niche overlap potentiates the use of endophytes in biocontrol of a tree disease. BioControl.

[7] Oliva J, Ridley M, Redondo MA, Caballol M (2021). Competitive exclusion amongst endophytes determines shoot blight severity on pine. Funct Ecol.

[8] Mandyam K, Jumpponen A (2005). Seeking the elusive function of the root-colonizing dark septate endophytic fungi. Stud Mycol.

[9] Ridout M, Newcombe G (2015). The frequency of modification of *Dothistroma* pine needle blight severity by fungi within the native range. For Ecol Manag.

[10] Miller JD, Sumarah MW, Adams GW (2008). Effect of a rugulosin-producing endophyte in *Picea glauca* on *Choristoneura fumiferana*. J Chem Ecol.

[11] Calhoun LA, Findlay JA, Miller JD, Whitney NJ (1992). Metabolites toxic to spruce budworm from balsam fir needle endophytes. Mycol Res.

[12] Miller JD, Mackenzie S, Foto M, Adams GW, Findlay JA (2002). Needles of white spruce inoculated with rugulosin-producing endophytes contain rugulosin reducing spruce budworm growth rate. Mycol Res.

[13] Terhonen E, Blumenstein K, Kovalchuk A, Asiegbu FO (2019). Forest tree microbiomes and associated fungal endophytes: functional roles and impact on Forest health. Forests.

[14] Slippers B, Wingfield MJ (2007). *Botryosphaeriaceae* as endophytes and latent pathogens of woody plants: diversity, ecology and impact. Fungal Biol Rev.

[15] Mehl JWM, Slippers B, Roux J, Wingfield MJ, Gonthier P, Nicolotti G (2013). Cankers and other diseases caused by the *Botryosphaeriaceae*. Infectious Forest diseases.

[16] Garbelotto M, Gonthier P (2013). Biology, Epidemiology, and Control of *Heterobasidion* Species Worldwide.

[17] Redfern BD, Stenlid J (1998). Spore dispersal and infection *Heterobasidion annosum.* Ecology, Impact and Control.

[18] Durodola B, Blumenstein K, Terhonen E. Genetic variation of *Picea abies* in response to the artificial inoculation of *Heterobasidion parviporum*. Eur J For Res. 2023; 10.1007/s10342-023-01534-3. 10.1007/s10342-023-01534-3 PMC9880357 36721489

[19] Cordier T, Robin C, Capdevielle X, Desprez-Loustau ML, Vacher C (2012). Spatial variability of phyllosphere fungal assemblages: genetic distance predominates over geographic distance in a European beech stand (*Fagus sylvatica*). Fungal Ecol.

[20] Bálint M, Tiffin P, Hallström B, O’Hara RB, Olson MS, Fankhauser JD, Piepenbring M, Schmitt I (2013). Host genotype shapes the foliar fungal microbiome of balsam poplar (*Populus balsamifera*). PLoS One.

[21] Rajala T, Velmala SM, Tuomivirta T, Haapanen M, Müller M, Pennanen T (2013). Endophyte communities vary in the needles of Norway spruce clones. Fungal Biol.

[22] Qian X, Duan T, Sun X, Zheng Y, Wang Y, Hu M, Yao H, Ji N, Lv P, Chen L, Shi M (2018). Host genotype strongly influences phyllosphere fungal communities associated with *Mussaenda pubescens* var. alba (*Rubiaceae*). Fungal Ecol.

[23] Terhonen E, Keriö S, Sun H, Asiegbu FO (2014). Endophytic fungi of Norway spruce roots in boreal pristine mire drained peatland and mineral soil and their inhibitory effect on *Heterobasidion parviporum* in vitro. Fungal Ecol.

[24] Terhonen E, Sipari N, Asiegbu FO (2016). Inhibition of phytopathogens by fungal root endophytes of Norway spruce. Biol Control.

[25] Tellenbach C, Sumarah MW, Grünig CR, Miller DJ (2013). Inhibition of *Phytophthora* species by secondary metabolites produced by the dark septate endophyte *Phialocephala europaea*. Fungal Ecol.

[26] Tedersoo L, Bahram M, Põlme S, Kõljalg U, Yorou NS, Wijesundera R, Ruiz LV, Vasco-Palacios AM, Thu PQ, Suija A, Smith ME, Sharp C, Saluveer E, Saitta A, Rosas M, Riit T, Ratkowsky D, Pritsch K, Põldmaa K, Abarenkov K (2014). Global diversity and geography of soil fungi. Science.

[27] Velmala SM, Rajala T, Haapanen M, Taylor AFS, Pennanen T (2013). Genetic host-tree effects on the ectomycorrhizal community and root characteristics of Norway spruce. Mycorrhiza.

[28] Elfstrand M, Zhou L, Baison J, Olson Å, Lundén K, Karlsson B, Wu HX, Stenlid J, García-Gil MR (2020). Genotypic variation in Norway spruce correlates to fungal communities in vegetative buds. Mol Ecol.

[29] Petrini O, Andrews JH, Hirano SS (1991). Fungal endophytes of tree leaves. Microbial ecology of leaves.

[30] Saikkonen K, Faeth SH, Helander M, Sullivan TJ (1998). Fungal endophytes: a continuum of interactions with host plants. Annu Rev Ecol Syst.

[31] Grünig CR, Duò A, Sieber TN (2006). Population genetic analysis of *Phialocephala fortinii* s.l. and *Acephala applanata* in two undisturbed forests in Switzerland and evidence for new cryptic species. Fungal Genet Biol.

[32] Stroheker S, Dubach V, Sieber TN (2018). Competitiveness of endophytic *Phialocephala fortinii* s.l. – Acephala applanata strains in Norway spruce roots. Fungal Biol.

[33] Jumpponen A, Trappe JM (1998). Dark septate endophytes: a review of facultative biotrophic root-colonizing fungi. New Phytol.

[34] Ahlich-Schlegel, K. Vorkommen und Charakterisierung von dunklen, septierten Hyphomyceten (DSH) in Gehölzwurzeln. Ph.D. thesis 1997, Department of Forest Sciences, Forest Pathology and Dendrology, Swiss Federal Institute of Technology, Zürich, Switzerland.

[35] Alberton O, Kuyper TW, Summerbell RC (2010). Dark septate root endophytic fungi increase growth of scots pine seedlings under elevated CO_2_ through enhanced nitrogen use efficiency. Plant Soil.

[36] Wang K, Wen Z, Asiegbu FO (2022). The dark septate endophyte *Phialocephala sphaeroides* suppresses conifer pathogen transcripts and promotes root growth of Norway spruce. Tree Physiol.

[37] Wen Z, Terhonen E, Asiegbu FO. The dark septate endophyte *Phialocephala sphaeroides* confers growth fitness benefits and mitigates pathogenic effects of *Heterobasidion* on Norway spruce, Tree Physiol, Volume 42, Issue 4, April 2022, Pages 891–906, 10.1093/treephys/tpab147. 10.1093/treephys/tpab147 PMC9000907 34791486

[38] Terhonen E, Langer G, Bußkamp J, Rӑscuţoi D, Blumenstein K (2019). Low water availability increases necrosis in *Picea abies* after artificial inoculation with fungal root rot pathogens *Heterobasidion parviporum* and *Heterobasidion annosum*. Forests.

[39] Yeoh XH-Y, Durodola B, Blumenstein K, Terhonen E (2021). Drought Stress Described by Transcriptional Responses of *Picea abies* (L.) H. Karst. under Pathogen *Heterobasidion parviporum* Attack. Forests.

[40] Desprez-Loustau M-L, Robin C, Reynaud G, Déqué M, Badeau V, Piou D, Husson C, Marçais B (2007). Simulating the effects of a climate-change scenario on the geographical range and activity of forest-pathogenic fungi. Can J Plant Pathol.

[41] Terhonen E, Oskay F, Kasanen R (2023). Editorial: the effect of mycobiomes on health of forest trees. Front Microbiol.

[42] Piri T, Kaitera J. Ylikiimingissä tehtiin tähän mennessä pohjoisin tyvitervastautihavainto. In: Terhonen, E. & Melin, M. (edit.) 2023; Metsätuhot vuonna 2022. [Forest damages in 2022]. Luonnonvara- ja biotalouden tutkimus 48/2023. Luonnonvarakeskus Helsinki p 18–20.

[43] Linnakoski R, Forbes KM, Wingfield MJ, Pulkkinen P, Asiegbu FO (2017). Testing projected climate change conditions on the *Endoconidiophora polonica* / Norway spruce Pathosystem shows fungal strain specific effects. Front Plant Sci.

[44] Wingfield MJ, Brockerhoff EG, Wingfield BD, Slippers B (2015). Planted forest health: the need for a global strategy. Science.

[45] Rodriguez RJ, White JF, Arnold AE, Redman RS (2009). Fungal endophytes: diversity and functional roles. New Phytol.

[46] Pennanen T, Heiskanen J, Korkama T (2005). Dynamics of ectomycorrhizal fungi and growth of Norway spruce seedlings after planting on a mounded forest clearcut. For Ecol Manag.

[47] Chang S, Puryear J, Cairney JA (1993). Simple and efficient method for isolating RNA from pine trees. Plant Mol Biol Report.

[48] Magoč T, Steven L, Salzberg. (2011). FLASH: fast length adjustment of short reads to improve genome assemblies. Bioinformatics.

[49] Bolyen E, Rideout JR, Dillon MR, Bokulich NA, Abnet CC, Al-Ghalith GA, Alexander H, Alm EJ, Arumugam M, Asnicar F, Bai Y, Bisanz JE, Bittinger K, Brejnrod A, Brislawn CJ, Brown CT, Callahan BJ, Caraballo-Rodríguez AM, Chase J, Cope EK, Da Silva R, Diener C, Dorrestein PC, Douglas GM, Durall DM, Duvallet C, Edwardson CF, Ernst M, Estaki M, Fouquier J, Gauglitz JM, Gibbons SM, Gibson DL, Gonzalez A, Gorlick K, Guo J, Hillmann B, Holmes S, Holste H, Huttenhower C, Huttley GA, Janssen S, Jarmusch AK, Jiang L, Kaehler BD, Kang KB, Keefe CR, Keim P, Kelley ST, Knights D, Koester I, Kosciolek T, Kreps J, Langille MGI, Lee J, Ley R, Liu YX, Loftfield E, Lozupone C, Maher M, Marotz C, Martin BD, McDonald D, McIver LJ, Melnik AV, Metcalf JL, Morgan SC, Morton JT, Naimey AT, Navas-Molina JA, Nothias LF, Orchanian SB, Pearson T, Peoples SL, Petras D, Preuss ML, Pruesse E, Rasmussen LB, Rivers A, Robeson MS, Rosenthal P, Segata N, Shaffer M, Shiffer A, Sinha R, Song SJ, Spear JR, Swafford AD, Thompson LR, Torres PJ, Trinh P, Tripathi A, Turnbaugh PJ, Ul-Hasan S, van der Hooft JJJ, Vargas F, Vázquez-Baeza Y, Vogtmann E, von Hippel M, Walters W, Wan Y, Wang M, Warren J, Weber KC, Williamson CHD, Willis AD, Xu ZZ, Zaneveld JR, Zhang Y, Zhu Q, Knight R, Caporaso JG (2019). Reproducible, interactive, scalable and extensible microbiome data science using QIIME 2. Nat Biotechnol.

[50] Abarenkov Kessy, Zirk Allan, Piirmann Timo, Pöhönen Raivo, Ivanov Filipp, Nilsson R Henrik, Kõljalg Urmas. UNITE QIIME release for Fungi 2. Version 10.05.2021. UNITE Community. 2021; 10.15156/BIO/1264763.

[51] Royston P (1982). An extension of Shapiro and Wilk's W test for normality to large samples. Appl Stat.

[52] Oksanen J, Simpson G, Blanchet F, Kindt R, Legendre P, Minchin P, O'Hara R, Solymos P, Stevens M, Szoecs E, Wagner H, Barbour M, Bedward M, Bolker B, Borcard D, Carvalho G, Chirico M, De Caceres M, Durand S, Evangelista H, FitzJohn R, Friendly M, Furneaux B, Hannigan G, Hill M, Lahti L, McGlinn D, Ouellette M, Ribeiro Cunha E, Smith T, Stier A, Ter Braak C, Weedon J. _vegan: Community Ecology Package_. R package version 2.6–4. 2022; https://CRAN.R-project.org/package=vegan.

[53] R Core Team. R: a language and environment for statistical computing. R Foundation for statistical computing. Vienna, Austria; 2023. https://www.R-project.org/

[54] Wickham H (2016). ggplot2: elegant graphics for data analysis.

[55] Foster ZSL, Sharpton TJ, Grünwald NJ (2017). Metacoder: an R package for visualization and manipulation of community taxonomic diversity data. PLoS Comput Biol.

[56] Põlme S, Abarenkov K, Henrik Nilsson R, Lindahl BD, Clemmensen KE, Kauserud H, Nguyen N, Kjøller R, Bates ST, Baldrian P, Frøslev TG, Adojaan K, Vizzini A, Suija A, Pfister D, Baral H-O, Järv H, Madrid H, Nordén J, Tedersoo L (2020). FungalTraits: a user-friendly traits database of fungi and fungus-like stramenopiles. Fungal Divers.

[57] Cáceres MD, Legendre P, Moretti M (2010). Improving Indicator species analysis by combining groups of sites. Oikos..

[58] Mantel NA (1967). The detection of disease clustering and a generalized regression approach. Cancer Res.

[59] Dray S, Dufour A (2007). The ade4 package: implementing the duality diagram for ecologists. J Stat Softw.

[60] Keriö S, Terhonen E, LeBoldus JM (2020). Safe DNA-extraction protocol suitable for studying tree-fungus interactions. Bio Protoc.

[61] White T, Bruns T, Lee S, Taylor J, Innis MA, Gelfand DH, Sninsky JJ, White TJ (1990). Amplification and direct sequencing of fungal ribosomal-RNA genes for phylogenetics. PCR protocols: a guide to methods and applications.

[62] Gardes M, Bruns TD (1993). ITS primers with enhanced specificity for higher fungi and basidiomycetes: application to identification of mycorrhizae and rusts. Mol Ecol.

[63] Marčiulynas A, Marčiulynienė D, Mishcherikova V, Franić I, Lynikienė J, Gedminas A, Menkis A (2022). High variability of fungal communities associated with the functional tissues and rhizosphere soil of *Picea abies* in the southern Baltics. Forests.

[64] Eusemann P, Schnittler M, Nilsson RH, Jumpponen A, Dahl MB, Würth DG, Buras A, Wilmking M, Unterseher M (2016). Habitat conditions and phenological tree traits overrule the influence of tree genotype in the needle mycobiome– *Picea glauc*a system at an arctic treeline ecotone. New Phytol.

[65] Redondo MA, Oliva J, Elfstrand M, Boberg J, Capador-Barreto HD, Karlsson B, Berlin A (2022). Host genotype interacts with aerial spore communities and influences the needle mycobiome of Norway spruce. Environ Microbiol.

[66] Menkis A, Marčiulynas A, Gedminas A, Lynikienė J, Povilaitienė A (2015). High-throughput sequencing reveals drastic changes in fungal communities in the Phyllosphere of Norway spruce (*Picea abies*) following invasion of the spruce bud scale (*Physokermes piceae*). Microb Ecol.

[67] Nguyen D, Boberg J, Ihrmark K, Stenström E, Stenlid J (2016). Do foliar fungal communities of Norway spruce shift along a tree species diversity gradient in mature European forests?. Fungal Ecol.

[68] Arnold AE, Lutzoni F (2007). Diversity and host range of foliar fungal endophytes: are tropical leaves biodiversity hotspots?. Ecology.

[69] Romeralo C, Martín-García J, Martínez-Álvarez P, Muñoz-Adalia EJ, Gonçalves DR, Torres E, Witzell J, Diez JJ (2022). Pine species determine fungal microbiome composition in a common garden experiment. Fungal Ecol.

[70] Schönrogge K, Gibbs M, Oliver A, Cavers S, Gweon HS, Ennos RA, Cottrell J, Iason GR, Taylor J (2022). Environmental factors and host genetic variation shape the fungal endophyte communities within needles of scots pine (*Pinus sylvestris*). Fungal Ecol.

[71] U’Ren JM, Lutzon F, Miadlikowska J, Zimmerman NB, Carbone I, May G, Arnold AE (2019). Host availability drives distributions of fungal endophytes in the imperilled boreal realm. Nat Ecol Evol.

[72] Alster CJ, Allison SD, Johnson NG, Glassman SI, Treseder KK (2021). Phenotypic plasticity of fungal traits in response to moisture and temperature. ISME Commun.

[73] Wrzosek M, Ruszkiewicz-Michalska M, Sikora K, Damszel M, Sierota Z (2017). The plasticity of fungal interactions. Mycol Prog.

[74] Cregger MA, Veach AM, Yang ZK, Crouch MJ, Vilgalys R, Tuskan GA, Schadt CW (2018). The *Populus* holobiont: dissecting the effects of plant niches and genotype on the microbiome. Microbiome.

[75] Horton MW, Bodenhausen N, Beilsmith K, Meng D, Muegge BD, Subramanian S, Vetter MM, Vilhjálmsson BJ, Nordborg M, Gordon JI, Bergelson J (2014). Genome-wide association study of *Arabidopsis thaliana* leaf microbial community. Nat Commun.

[76] Martínez-Arias C, Sobrino-Plata J, Macaya-Sanz D, Aguirre NM, Collada C, Gil L, Martín JA, Rodríguez-Calcerrada J (2020). Changes in plant function and root mycobiome caused by flood and drought in a riparian tree. Tree Physiol.

[77] Sapkota R, Knorr K, Jørgensen LN, O’Hanlon KA, Nicolaisen M (2015). Host genotype is an important determinant of the cereal phyllosphere mycobiome. New Phytol.

[78] Castaño C, Suarez-Vidal E, Zas R, Bonet JA, Oliva J, Sampedro L (2023). Ectomycorrhizal fungi with hydrophobic mycelia and rhizomorphs dominate in young pine trees surviving experimental drought stress. Soil Biol Biochem.

[79] Gehring CA, Sthultz CM, Flores-Rentería L, Whipple AV, Whitham TG (2017). Tree genetics defines fungal partner communities that may confer drought tolerance. Proc Natl Acad Sci U S A.

[80] Hilszczańska D, Sierota Z (2013). Persistence of ectomycorrhizas by *Thelephora terrestris* on outplanted scots pine seedlings. Acta Mycol.

[81] Gehring C, Sevanto S, Patterson A, Ulrich DEM, Kuske CR (2020). Ectomycorrhizal and dark septate fungal associations of pinyon pine are differentially affected by experimental drought and warming. Front Plant Sci.

[82] Köhler J, Yang N, Pena R, Raghavan V, Polle A, Meier IC (2018). Ectomycorrhizal fungal diversity increases phosphorus uptake efficiency of European beech. New Phytol.

[83] Liu N, Jacquemyn H, Liu Q, Shao S-C, Ding G, Xing X (2022). Effects of a dark septate fungal endophyte on the growth and physiological response of seedlings to drought in an epiphytic orchid. Front Microbiol.

[84] Wagner K, Krause K, Gallegos-Monterrosa R, Sammer D, Kovács ÁT, Kothe E (2019). The Ectomycorrhizospheric habitat of Norway spruce and *Tricholoma vaccinum*: promotion of plant growth and fitness by a rich microorganismic community. Front Microbiol.

[85] Boczoń A, Hilszczańska D, Wrzosek M, Szczepkowski A, Sierota Z (2021). Drought in the forest breaks plant–fungi interactions. Eur J For Res.

[86] Mohan JE, Cowden CC, Baas P, Dawadi A, Frankson PT, Helmick K, Hughes E, Khan S, Lang A, Machmuller M, Taylor M, Witt CA (2014). Mycorrhizal fungi mediation of terrestrial ecosystem responses to global change: Mini-review. Fungal Ecol.

[87] Baldrian P, López-Mondéjar R, Kohout P. Forest microbiome and global change. Nat Rev Microbiol. 2023; 10.1038/s41579-023-00876-4. 10.1038/s41579-023-00876-4 36941408

[88] Anderegg WRL, Trugman AT, Badgley G, Anderson CM, Bartuska A, Ciais P, Cullenward D, Field CB, Freeman J, Goetz SJ, Hicke JA, Huntzinger D, Jackson RB, Nickerson J, Pacala S, Randerson JT (2020). Climate-driven risks to the climate mitigation potential of forests. Science.

[89] Bastida F, López-Mondéjar R, Baldrian P, Andrés-Abellán M, Jehmlich N, Torres IF, García C, López-Serrano FR (2019). When drought meets forest management: effects on the soil microbial community of a holm oak forest ecosystem. Sci Total Environ.

[90] Márquez LM, Redman RS, Rodriguez RJ, Roossinck MJ (2007). A virus in a fungus in a plant: three-way symbiosis required for thermal tolerance. Science.

[91] Redman RS, Sheehan KB, Stout RG, Rodriguez RJ, Henson JM (2002). Thermotolerance generated by plant/fungal Symbiosis. Science.

[92] Redman RS, Kim YO, Woodward CJDA, Greer C, Espino L, Doty SL, Rodriguez RJ (2011). Increased fitness of Rice plants to abiotic stress via habitat adapted Symbiosis: a strategy for mitigating impacts of climate change. PLoS One.

[93] Schulz B, Römmert A, Dammann U, Aust H, Strack D (1999). The endophyte-host interaction: a balanced antagonism?. Mycol Res.

[94] Kosawang C, Amby DB, Bussaban B, McKinney LV, Xu J, Kjær ED, Collinge DB, Nielsen LR (2018). Fungal communities associated with species of *Fraxinus* tolerant to ash dieback, and their potential for biological control. Fungal Biol.

[95] Berthelot C, Leyval C, Chalot M, Blaudez D (2019). Interactions between dark septate endophytes, ectomycorrhizal fungi and root pathogens in vitro. FEMS Microbiol Lett.

[96] Blumenstein K, Bußkamp J, Langer GJ, Schlößer R, Parra Rojas NM, Terhonen E (2021). *Sphaeropsis sapinea* and associated endophytes in scots pine: interactions and effect on the host under variable water content. Front For Glob Change.

[97] Arnold AE, Mejía LC, Kyllo D, Rojas EI, Maynard Z, Robbins N, Herre EA (2003). Fungal endophytes limit pathogen damage in a tropical tree. Proc Natl Acad Sci.

[98] Martínez-Arias C, Sobrino-Plata J, Gil L, Rodríguez-Calcerrada J, Martín JA (2021). Priming of plant defenses against *Ophiosoma novo-ulmi* by elm (*Ulmus minor* mill.) fungal endophytes. J Fungi.

[99] Martínez-Arias C, Sobrino-Plata J, Medel D, Gil L, Martín JA, Rodríguez-Calcerrada J (2021). Stem endophytes increase root development, photosynthesis, and survival of elm plantlets (*Ulmus minor* mill.). J Plant Physiol.

[100] Martínez-Arias C, Sobrino-Plata J, Ormeño-Moncalvillo S, Gil L, Rodríguez-Calcerrada J, Martín JA (2021). Endophyte inoculation enhances *Ulmus minor* resistance to Dutch elm disease. Fungal Ecol.

[101] Würth DG, Dahl MB, Trouillier M, Wilmking M, Unterseher M, Scholler M, Sørensen S, Mortensen M, Schnittler M (2019). The needle mycobiome of Picea glauca – a dynamic system reflecting surrounding environment and tree phenological traits. Fungal Ecol.

[102] Shi L, Dossa GGO, Paudel E, Zang H, Xu J, Harrison RD (2019). Changes in fungal communities across a Forest disturbance gradient. Appl Environ Microbiol.

