# Supplementary material for: Beyond the surface: exploring the mycobiome of Norway spruce under drought stress and with Heterobasidion parviporum
Source: BMC Microbiol. 2023 Nov 17;23:350. doi: 10.1186/s12866-023-03099-y (PMC10655427; doi:10.1186/s12866-023-03099-y)
Supplement: Supplementary file 1 — Additional file 1. [file 12866_2023_3099_MOESM1_ESM.docx]

**Beyond the surface: Exploring the mycobiome of Norway spruce under abiotic stress and with *Heterobasidion parviporum***

Blessing Durodola^1,2^, Kathrin Blumenstein^1,3^, Adedolapo Akinbobola^1^, Anna Kolehmainen^1,4^, Victor Chano^2^, Oliver Gailing^2^, and Eeva Terhonen^1,5^

**Supplementary figures**

| **A**  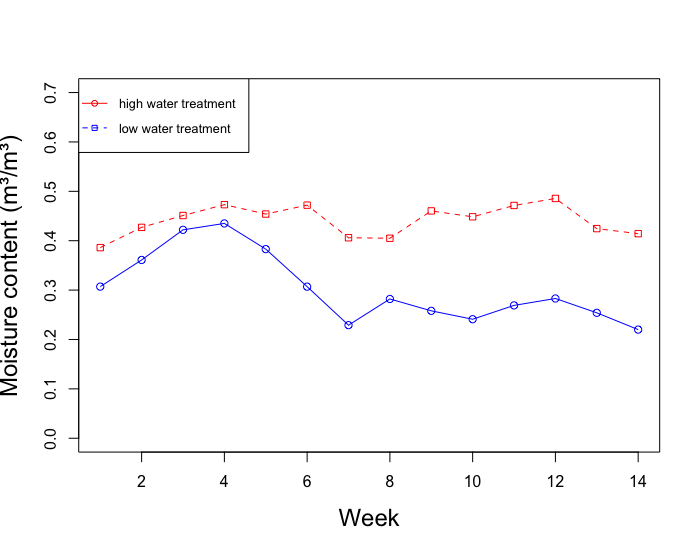 | **B**  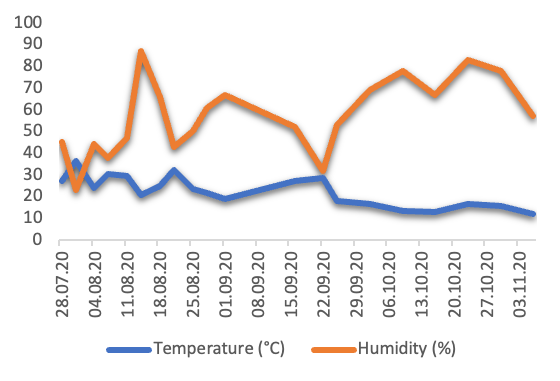 |
| --- | --- |

**Fig. S1** Abiotic parameters measured during the experiment. **A)** soil moisture, **B)** atmospheric temperature and humidity.


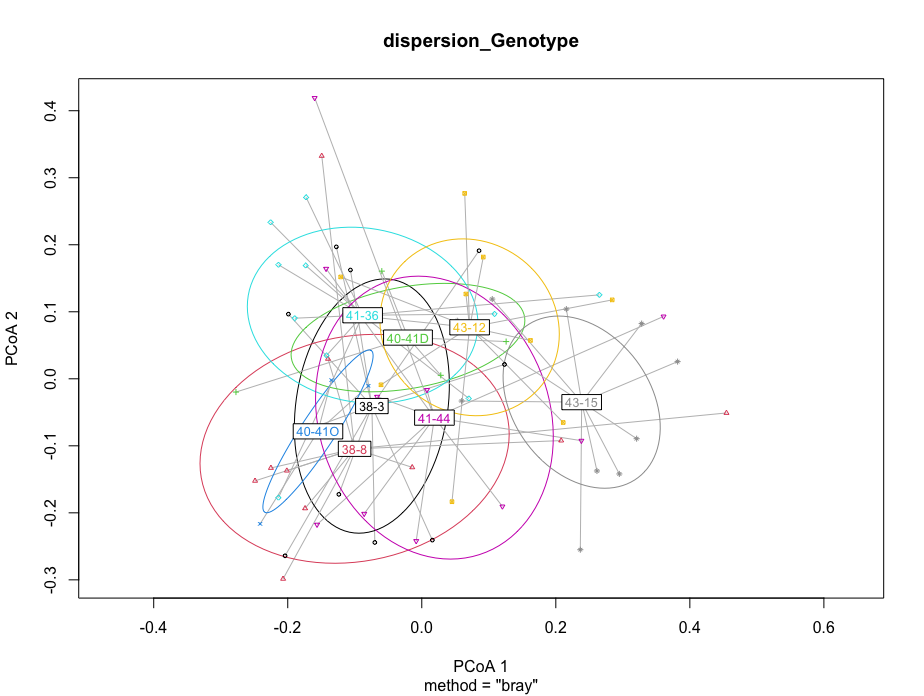


**Fig. S2** Fungal communities among phloem genotypes based on Bray-Curtis.


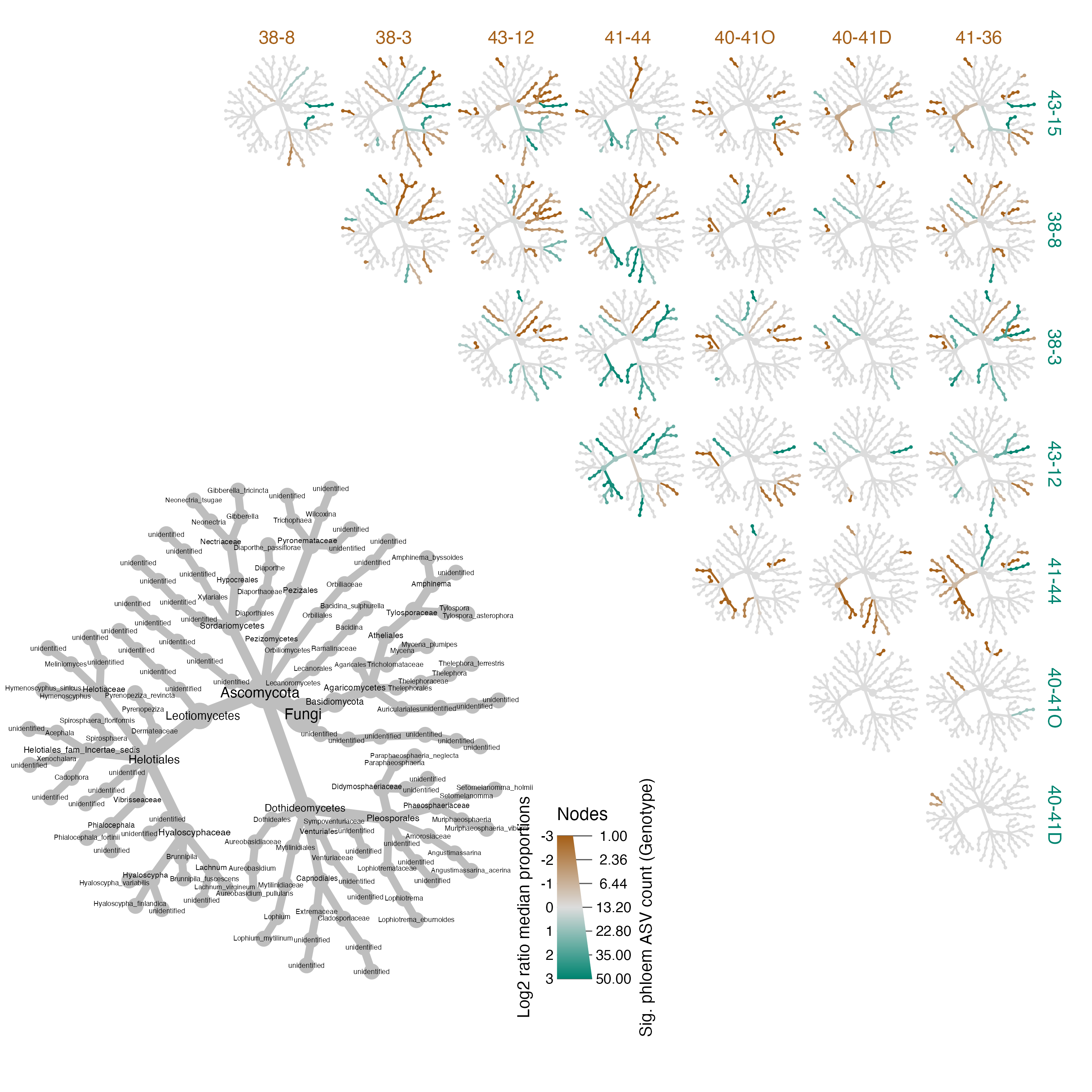
 **Fig. S3** Pairwise comparisons of the phloem mycobiome between the different genotypes. The colour of each taxon represents the log-2 ratio of median proportions of phloem ASV counts observed at each genotype. Taxa-coloured green are enriched in the genotypes shown in the rows, and those coloured cyan are enriched in the genotypes shown in the columns. The grey tree on the lower left serves as a key for the smaller unlabelled trees.


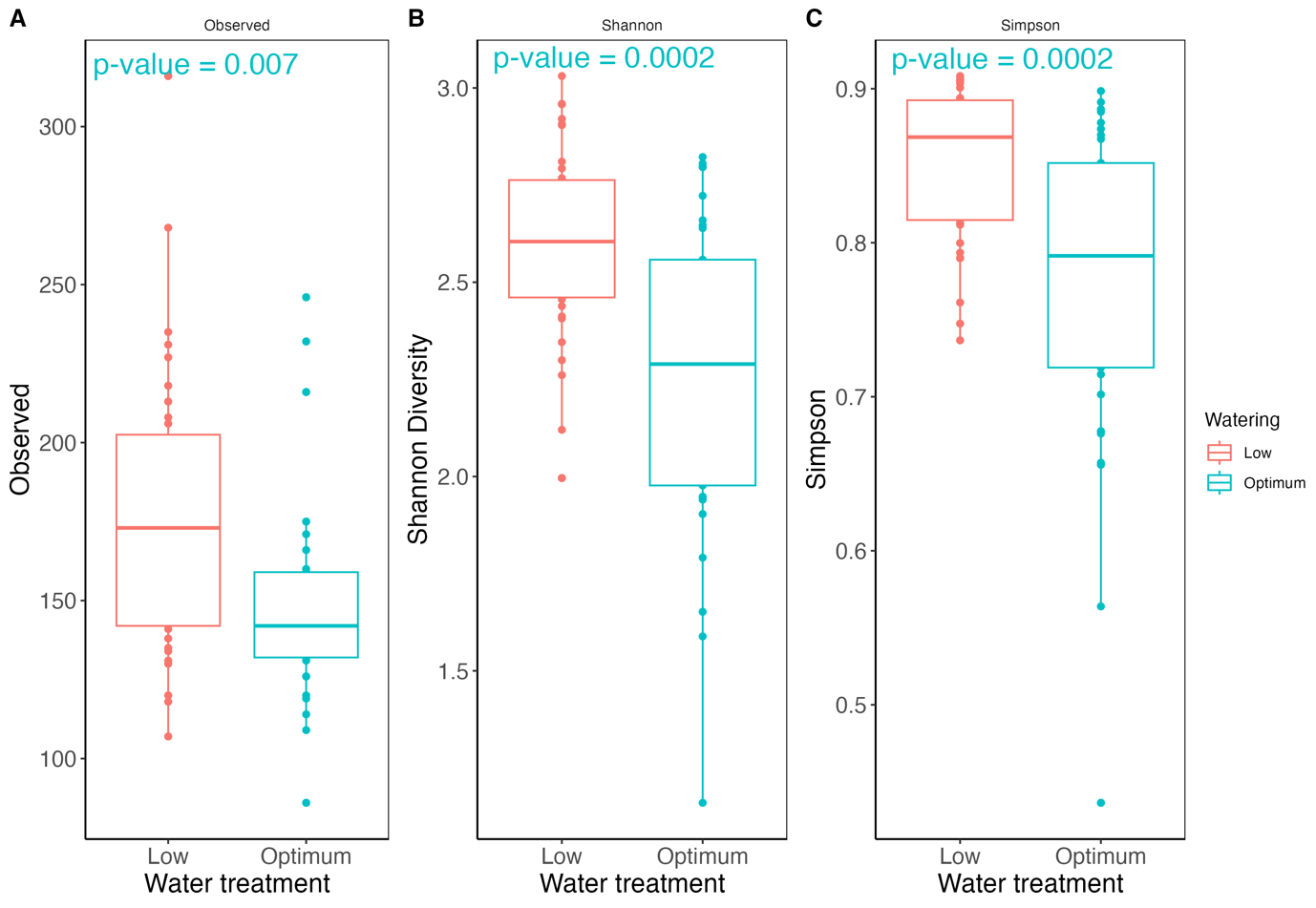


**Fig. S4** Alpha diversity for water treatments. **A)** Observed amplicon sequence variants, B**)** Shannon diversity index, **C)** Simpson index.


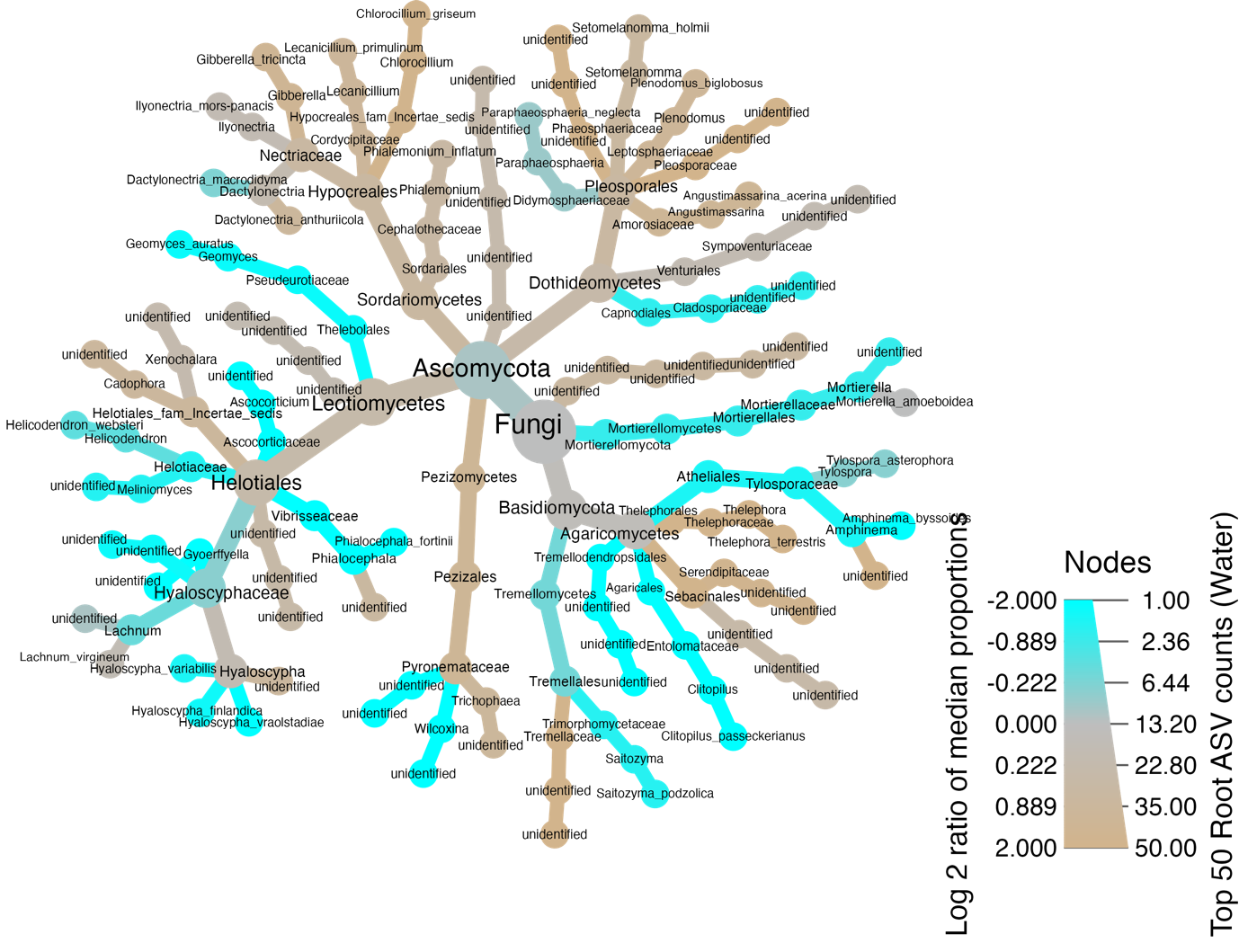


**Fig. S5** Root mycobiome abundance according to watering groups. The colour of each taxon represents the log-2 ratio of median proportions of phloem ASV counts observed at each water treatment category. Taxa-coloured tan are more abundant in the optimum watering category, while taxa-coloured cyan are more abundant in low-water treated plants.

**
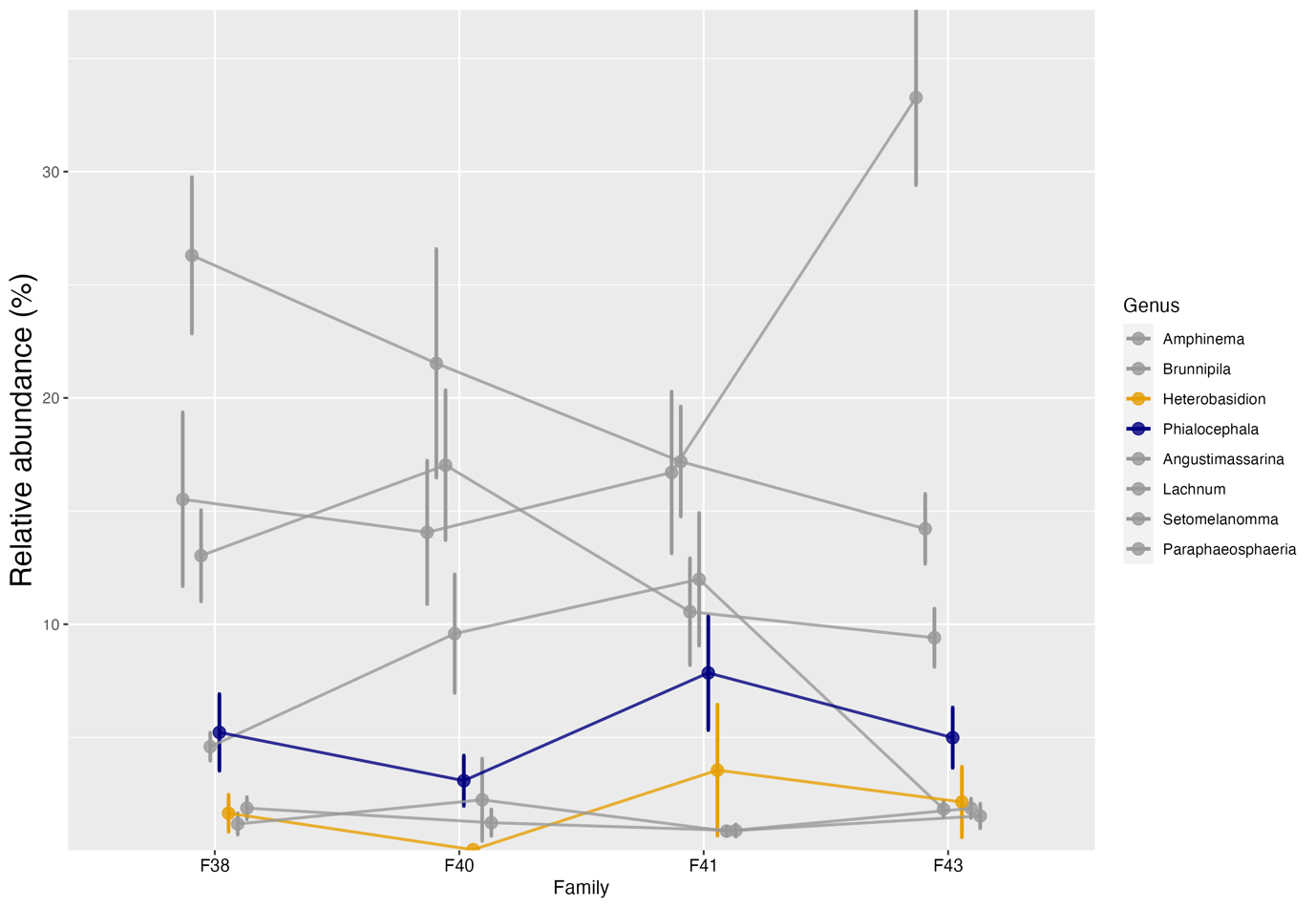
Fig. S6** Relative abundance of taxa of the top eight fungi among families in the presence of *H*. *parviporum* highlighting only the pattern between Phialocephala and Heterobasidion genera. Connecting lines are included to visualize common patterns.

**Supplementary tables**

**Table S1** Information on phloem plant materials genotype and treatment.

| **Sample ID** | **Family** | **Genotype** | **Water-treatment** | **Inoculation treatment** |
| --- | --- | --- | --- | --- |
| D21 | F38 | 38-3A | Drought | Hpa2 |
| D37 | F38 | 38-3B | Drought | Hpa1 |
| D51 | F38 | 38-3J | Drought | Non-treated |
| D52 | F38 | 38-3D | Drought | Hpa1 |
| D53 | F38 | 38-3F | Drought | Hpa2 |
| D54 | F38 | 38-3G | Drought | Hpa2 |
| D55 | F38 | 38-3E | Drought | Hpa1 |
| D56 | F38 | 38-3C | Drought | Control |
| D57 | F38 | 38-3I | Drought | Control |
| D58 | F38 | 38-3H | Drought | Control |
| D11 | F38 | 38-8J | Optimum | Non-treated |
| D12 | F38 | 38-8F | Optimum | Hpa2 |
| D13 | F38 | 38-8G | Optimum | Hpa1 |
| D14 | F38 | 38-8C | Optimum | Control |
| D15 | F38 | 38-8E | Optimum | Hpa1 |
| D16 | F38 | 38-8A | Optimum | Control |
| D17 | F38 | 38-8I | Optimum | Hpa1 |
| D18 | F38 | 38-8H | Optimum | Control |
| D19 | F38 | 38-8B | Optimum | Hpa2 |
| D20 | F38 | 38-8D | Optimum | Hpa2 |
| D25 | F40 | 40-41G | Optimum | Non-treated |
| D26 | F40 | 40-41A | Optimum | Non-treated |
| D27 | F40 | 40-41H | Drought | Non-treated |
| D28 | F40 | 40-41J | Optimum | Non-treated |
| D29 | F40 | 40-41B | Drought | Non-treated |
| D30 | F40 | 40-41E | Drought | Non-treated |
| D85 | F40 | 40-41I | Drought | Non-treated |
| D34 | F41 | 41-36D | Drought | Hpa2 |
| D35 | F41 | 41-36B | Drought | Hpa1 |
| D38 | F41 | 41-36F | Drought | Control |
| D40 | F41 | 41-36I | Drought | Non-treated |
| D41 | F41 | 41-36C | Drought | Hpa1 |
| D42 | F41 | 41-36G | Drought | Control |
| D43 | F41 | 41-36A | Drought | Control |
| D44 | F41 | 41-36H | Drought | Hpa1 |
| D45 | F41 | 41-36E | Drought | Hpa2 |
| D46 | F41 | 41-36J | Drought | Hpa2 |
| D24 | F41 | 41-44I | Optimum | Control |
| D36 | F41 | 41-44C | Optimum | Hpa2 |
| D59 | F41 | 41-44H | Optimum | Control |
| D60 | F41 | 41-44A | Optimum | Non-treated |
| D61 | F41 | 41-44D | Optimum | Hpa1 |
| D62 | F41 | 41-44J | Optimum | Hpa1 |
| D63 | F41 | 41-44B | Optimum | Control |
| D64 | F41 | 41-44G | Optimum | Hpa2 |
| D65 | F41 | 41-44F | Optimum | Hpa2 |
| D66 | F41 | 41-44E | Optimum | Hpa1 |
| D22 | F43 | 43-12A | Drought | Hpa2 |
| D23 | F43 | 43-12F | Drought | Control |
| D31 | F43 | 43-12J | Drought | Non-treated |
| D32 | F43 | 43-12E | Drought | Control |
| D33 | F43 | 43-12D | Drought | Hpa2 |
| D39 | F43 | 43-12H | Drought | Control |
| D47 | F43 | 43-12C | Drought | Hpa1 |
| D48 | F43 | 43-12B | Drought | Hpa1 |
| D49 | F43 | 43-12G | Drought | Hpa2 |
| D50 | F43 | 43-12I | Drought | Hpa1 |
| D10 | F43 | 43-15H | Optimum | Hpa2 |
| D1 | F43 | 43-15F | Optimum | Hpa2 |
| D2 | F43 | 43-15E | Optimum | Control |
| D3 | F43 | 43-15G | Optimum | Hpa1 |
| D4 | F43 | 43-15A | Optimum | Non-treated |
| D5 | F43 | 43-15C | Optimum | Control |
| D6 | F43 | 43-15I | Optimum | Hpa1 |
| D7 | F43 | 43-15B | Optimum | Hpa1 |
| D8 | F43 | 43-15D | Optimum | Control |
| D9 | F43 | 43-15J | Optimum | Hpa2 |

**Table S2** Abundance of dark septate endophyte (DSE) and Ectomycorrhizal (ECM) fungi in different root water treatments.

| **Phylum** | **Genera** | **Optimum** | **Low** | **Relative** | **Fungi type** |
| --- | --- | --- | --- | --- | --- |
|  |  |  |  | **Abundance (%)** |  |
| Ascomycota | *Phialocephala* | 87491 | 144730 | 10.1 | DSE |
| Ascomycota | *Cadophora* | 2164 | 2015 | 0.2 | DSE |
| Ascomycota | *Exophiala* | 696 | 485 | 0.1 | DSE |
| Ascomycota | *Acephala* | 397 | 812 | 0.1 | DSE |
| Ascomycota | *Gyoerffyella* | 101 | 1029 | 0.0493 | DSE |
| Ascomycota | *Cladophialophora* | 36 | 34 | 0.0031 | DSE |
| Ascomycota | *Capronia* | 0 | 63 | 0.0028 | DSE |
| Ascomycota | *Phialophora* | 0 | 3 | 0.0001 | DSE |
| Basidiomycota | *Amphinema* | 224396 | 341543 | 24.7 | ECM |
| Basidiomycota | *Thelephora* | 211607 | 11165 | 9.7 | ECM |
| Ascomycota | *Trichophaea* | 173817 | 108305 | 12.3 | ECM |
| Ascomycota | *Wilcoxina* | 57384 | 118050 | 7.7 | ECM |
| Basidiomycota | *Tylospora* | 3430 | 1244 | 0.2 | ECM |
| Basidiomycota | *Inocybe* | 79 | 5 | 0.0037 | ECM |
| Basidiomycota | *Hygrophorus* | 10 | 0 | 0.0004 | ECM |
| Basidiomycota | *Tomentella* | 9 | 3 | 0.0005 | ECM |
| Basidiomycota | *Laccaria* | 5 | 0 | 0.0002 | ECM |
| Ascomycota | *Geopora* | 3 | 0 | 0.0001 | ECM |
| Ascomycota | *Cenococcum* | 0 | 24 | 0.0010 | ECM |
| Basidiomycota | *Rhizopogon* | 0 | 10 | 0.0004 | ECM |
| Basidiomycota | *Amanita* | 0 | 5 | 0.0002 | ECM |

**Table S3** Pairwise genetic distance (below the diagonal) and pairwise distance based on taxa abundance (above the diagonal) for the genotypes used in both water regimes (optimum watering and drought stress).

| *Optimum watering* | | | | |
| --- | --- | --- | --- | --- |
|  | **38-8** | **40-41** | **41-44** | **43-15** |
| **38-8** | -- | 43225.39 | 46753.1 | 41050.17 |
| **40-41** | 217.01 | -- | 28012.91 | 20060.94 |
| **41-44** | 214.51 | 199.49 | -- | 22450.02 |
| **43-15** | 215.6 | 218.11 | 215.47 | -- |
| *Low watering* | | | | |
|  | **38-3** | **40-41** | **41-36** | **43-12** |
| **38-3** | -- | 31795.45 | 40825.93 | 50635.47 |
| **40-41** | 220.26 | -- | 29683.51 | 31918.36 |
| **41-36** | 216.59 | 197.34 | -- | 38605.93 |
| **43-12** | 216.66 | 217.4 | 214.25 | -- |
